# Systematic review supports the role of DNA methylation in the pathophysiology of preeclampsia: a call for analytical and methodological standardization

**DOI:** 10.1186/s13293-020-00313-8

**Published:** 2020-07-06

**Authors:** A. Cirkovic, V. Garovic, J. Milin Lazovic, O. Milicevic, M. Savic, N. Rajovic, N. Aleksic, T. Weissgerber, A. Stefanovic, D. Stanisavljevic, N. Milic

**Affiliations:** 1 Institute for Medical Statistics and Informatics, Faculty of Medicine, University of Belgrade, Belgrade, Serbia; 2 Department of Internal Medicine, Division of Nephrology and Hypertension, Mayo Clinic, Rochester, MN USA; 3 Center for Molecular Biology, University of Vienna, Vienna, Austria; 4 Charité - Universitätsmedizin Berlin, Berlin Institute of Health, QUEST Center, Berlin, Germany; 5 Clinic for Gynecology and Obstetrics, Clinical Centre of Serbia, Belgrade, Serbia

**Keywords:** Epigenetics, Methylation, Preeclampsia, Meta-research

## Abstract

**Background:**

Studies have recently examined the role of epigenetic mechanisms in preeclampsia pathophysiology. One commonly examined epigenetic process is DNA methylation. This heritable epigenetic marker is involved in many important cellular functions. The aim of this study was to establish the association between DNA methylation and preeclampsia and to critically appraise the roles of major study characteristics that can significantly impact the association between DNA methylation and preeclampsia.

**Main body:**

A systematic review was performed by searching PubMed, Web of Science, and EMBASE for original research articles published over time, until May 31, 2019 in English. Eligible studies compared DNA methylation levels in pregnant women with vs. without preeclampsia. Ninety articles were included. Epigenome-wide studies identified hundreds of differentially methylated places/regions in preeclamptic patients. Hypomethylation was the predominant finding in studies analyzing placental tissue (14/19), while hypermethylation was detected in three studies that analyzed maternal white blood cells (3/3). In candidate gene studies, methylation alterations for a number of genes were found to be associated with preeclampsia. A greater number of differentially methylated genes was found when analyzing more severe preeclampsia (70/82), compared to studies analyzing less severe preeclampsia vs. controls (13/27). A high degree of heterogeneity existed among the studies in terms of methodological study characteristics including design (study design, definition of preeclampsia, control group, sample size, confounders), implementation (biological sample, DNA methylation method, purification of DNA extraction, and validation of methylation), analysis (analytical method, batch effect, genotyping, and gene expression), and data presentation (methylation quantification measure, measure of variability, reporting). Based on the results of this review, we provide recommendations for study design and analytical approach for further studies.

**Conclusions:**

The findings from this review support the role of DNA methylation in the pathophysiology of preeclampsia. Establishing field-wide methodological and analytical standards may increase value and reduce waste, allowing researchers to gain additional insights into the role of DNA methylation in the pathophysiology of preeclampsia.

## Introduction

Preeclampsia (PE) is a life-threatening condition, affecting 2 to 8% of all pregnancies worldwide [1–3]. It contributes significantly to maternal and fetal morbidity and mortality [4, 5]. Women with preeclampsia have higher risks for acute renal failure, cerebral bleeding, liver dysfunction, pulmonary edema, and disseminated intravascular coagulation [6]. Well-known risk factors for preeclampsia include antiphospholipid syndrome, previous preeclampsia, insulin-dependent diabetes, multiple pregnancy, nulliparity, family history of preeclampsia, obesity, age over 40 years, and preexisting hypertension [7, 8]. The exact etiology of PE, however, remains unknown. Study of PE causation is further complicated by the existence of heterogeneous forms of the disease. Preeclampsia is categorized as early or late onset based upon whether gestational age is prior to or after 34 weeks. Early-onset PE (EOPE), compared to late-onset PE (LOPE), is characterized by more severe disease, and commonly presented with severe placental dysfunction and adverse maternal and fetal outcomes, including intrauterine fetal growth restriction [9]. However, it remains poorly understood whether EOPE and LOPE are two different clinical entities that have different pathogenetic mechanisms or represent the same underlying condition of different severity.

Studies recently have examined the role of epigenetic mechanisms in PE pathophysiology. One commonly examined epigenetic process is DNA methylation, a covalent ligation of a methyl group to the C5 position of the cytosine in a CpG site of DNA by DNA methyltransferases. This heritable epigenetic marker is involved in many important cellular functions including embryonic development, transcription, chromatin structure, and X chromosome inactivation [10]. A number of studies have explored DNA methylation in PE. Earlier studies have reported global genome methylation profiles in PE [11–13] or have examined the methylation level of preselected candidate genes [14–16]. More recently, differentially methylated probes/regions (DMPs/DMRs) between PE versus controls were identified epigenome-wide using high-throughput platforms [17–19]. While these studies reported hundreds of DMPs and DMRs in the DNA methylome in PE, the results were inconsistent, likely reflecting design limitations, such as the small sample sizes and lack of standardization of the analytical approaches [20]. Moreover, independent replications of key findings were lacking, thus limiting the understanding of epigenetic programming in PE.

Recently published reviews [20–22] have attempted to establish the role of epigenetics in placental development and the etiology of PE, but they did not critically examine the experimental methodologies, closely linked to the accuracy of the results. There has been no systematic review conducted to summarize the relationship between DNA methylation and PE. The aim of this study was to synthesize the evidence regarding the association between DNA methylation and PE and to critically appraise the roles of major study characteristics that can significantly impact the association between DNA methylation and preeclampsia.

## Material and methods

The systematic review was performed in accordance with the Preferred Reporting Items for Systematic Reviews and Meta-Analyses (PRISMA) guidelines and MOOSE Guidelines for Meta-Analyses and Systematic Reviews of Observational Studies [23, 24].

### Study selection

Publications identified were evaluated for inclusion in the study in two phases, and all disagreements were resolved by discussion at each stage. Two reviewers (AC and OM) independently reviewed all titles and abstracts and selected the potentially relevant publications. Full-text copies of the selected publications were obtained and assessed independently by two reviewers according to the study inclusion criteria. We examined studies that compared DNA methylation levels among women who had PE and pregnant women that did not have PE. Studies were eligible for inclusion if DNA methylation was measured in both groups. We excluded studies that (i) investigated other outcomes, (ii) did not include comparisons between pregnant women with vs. without PE, (iii) examined populations other than those with PE, (iv) did not include women but included animals or cell lines, (v) assessed other epigenetic markers, (vi) were abstracts, or (vii) were not original articles. Discrepancies were resolved through a third reviewer.

In studies for which diagnostic criteria were listed in the paper or provided by the study authors, PE was diagnosed according to accepted guidelines (American College of Obstetricians and Gynecologists, International Society for the Study of Hypertension in Pregnancy, Report of the National High Blood Pressure Program Working Group) [25–27], or using equivalent criteria for hypertension and proteinuria appearing after 20 weeks of gestation.

Studies were stratified into one of the following subgroups:

1. More severe preeclampsia: early-onset preeclampsia (EOPE), severe preeclampsia (sPE)

2. Less severe preeclampsia: late-onset preeclampsia (LOPE), mild preeclampsia (mPE)

3. Not specified preeclampsia: studies without reported severity of preeclampsia

### Database search

Two biostatisticians with expertise in conducting systematic reviews and meta-analyses (NM, AC) developed the search strategy. A systematic review of all published peer-reviewed articles was performed through searches of PubMed, Web of Science (Wos), and EMBASE electronic databases over time, until May 31, 2019. Search queries differed according to the database, and keywords for the PubMed search were preeclampsia and (epigenetic or epigenetics or miRNA or microRNA or DNA methylation or DNA methylations or long non-coding RNA), for Wos: TS = *eclampsia and TS = (epigenetic* or microRNA or DNA methylation or gene imprinting or long non-coding RNA), and for EMBASE: preeclampsia and (epigenetics or microRNA or DNA methylation or genome imprinting or long untranslated RNA). Only publications in English were included. In addition, reference lists of articles identified through electronic retrieval were manually searched, as well as relevant reviews and editorials. Experts in the field were contacted to identify other potentially relevant articles.

### Article screening and selection

Two reviewers (AC, OM) independently evaluated the eligibility of all titles and abstracts. Studies were included in the full-text screening if either reviewer identified the study as being potentially eligible or if the abstract and title did not include sufficient information. Studies were eligible for full-text screening if they included comparisons of DNA methylation levels between women with PE and pregnant women that did not have PE. Preeclampsia included more severe, less severe, and not specified forms. The same reviewers independently performed full-text screening to select articles for inclusion according to the inclusion and exclusion criteria. Disagreements were resolved by consensus (AC, OM) or arbitration (NM, DS).

### Data abstraction and quality assessment

Two reviewers independently abstracted the following data: author(s), country of research, year of publication, study design, sample size, study population, preeclampsia definitions and type, inclusion and exclusion criteria, sample type and time of DNA collection, epigenetic approach, measures used for DNA methylation level quantification, DNA methylome presentation and reporting, and corresponding gene(s) (with the number of CpGs and region), as well as DNA purification, DNA methylome validation, genotyping, batch effect correction, and mRNA expression. Each reviewer independently evaluated the quality of selected manuscripts using an adapted version of the Newcastle-Ottawa tool for observational studies [28]. Independent reviewers used standardized forms and protocols when selecting and abstracting data. All details regarding study protocol and study characteristics are available at https://osf.io/dv85n/.

### Statistical analysis

Data are presented as averages with standard deviations (or standard errors) or medians with ranges for numerical variables. Categorical variables are presented as absolute and relative numbers. Over-representation analysis (ORA) was performed using the PANTHER web tool on the list of gene IDs extracted in the previous steps. The reference database of entities was the Gene Ontology (GO) molecular function complete database, while the reference list of genes was the PANTHER whole-genome list for Homo-sapiens. Fisher’s exact test was used with False Discovery Rate (FDR) to correct for multiple testing errors.

## Results

**Fig. 1:**
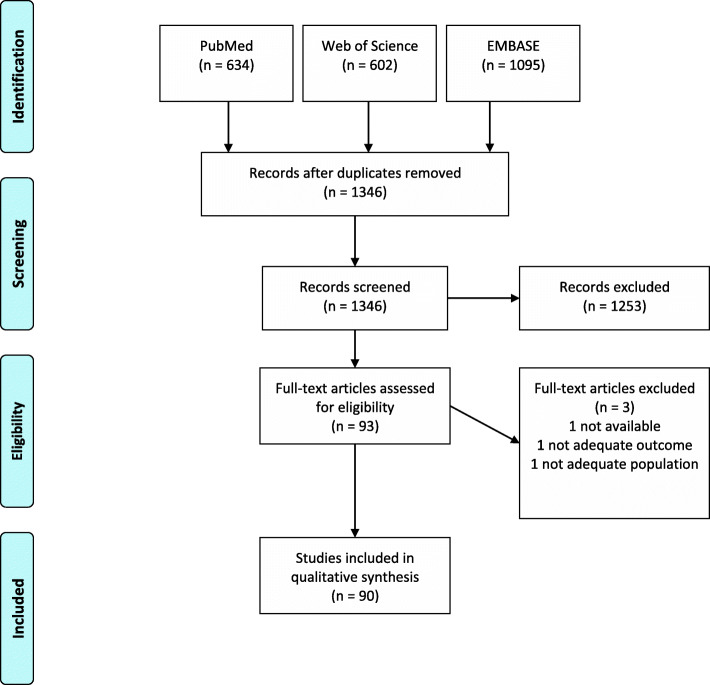
Flow diagram

We identified 1346 potentially eligible articles. Upon inspection of the titles and abstracts, 1253 articles were excluded as they were not original articles, were without PE as the outcome, did not compare PE and control groups, examined populations other than women (animals, cell lines), did not explore methylation levels, or were abstracts. Of the 93 full-text articles that were reviewed, 90 were selected for inclusion in the systematic review. The process of study selection through the different phases of a systematic review is presented in Fig. 1. All 90 full-text publications included in the systematic review are presented in detail in Additional file 1, which includes tables describing the summary characteristics for the included studies (Table S1), the diagnostic criteria for studies examining women with more severe and less severe forms of PE (Tables S2, S3), and exclusion criteria in the reviewed studies (Table S4).

### Global DNA methylation

Four studies examined the association between global DNA methylation and PE. Three studies assessed global DNA methylation in the placenta [11–13, 29], while one study assessed DNA methylation using both placenta and umbilical cord blood [29]. Increased global DNA methylation levels in both term and preterm PE placental samples compared to normotensive controls were found in one study [12]. Similarly, another study found that LINE-1 methylation levels were significantly higher in the EOPE placentas compared to normal controls [11]. One study reported no differences in global methylation levels in the placentas of PE and normotensive pregnant women [13]. The study that assessed DNA methylation both in the placental tissue and umbilical cord blood reported lower global methylation levels in placental samples of patients with PE compared to healthy controls, but not in the umbilical cord blood. The overall conclusion, regarding studies that examined global DNA methylation in the placental tissue, is that hyper-, hypo-, and no significant difference in DNA methylation levels were found in PE compared to pregnant women without PE.

### Epigenome-wide analysis

There were 30 studies using an epigenome-wide approach (EWAS) to search for differentially methylated sites associated with PE. Four of them used an EWAS approach only (two studies used the placental tissue, one used the maternal peripheral blood and placenta, and one used the umbilical cord blood and placenta), and 26 studies combined this technique with candidate gene analysis. Twenty-three studies analyzed epigenome-wide methylation levels in the placental tissue; two studies used the maternal peripheral blood (white blood cells—WBC), three studies used the umbilical cord blood, three studies used the omental fat arteries, one study looked at both placental tissue and maternal peripheral blood (WBC), and one study looked at both placental and umbilical cord blood cells (Table 1). All epigenome-wide studies, with the exception of two, reported significant methylation sites, including hyper- and hypomethylation, in patients with PE compared to pregnant women without PE (Table 1). In 14 of 19 studies, hypomethylation was found in the placental tissue, while in three studies, hypermethylation was detected in maternal WBC. Results from the umbilical cord blood studies found the opposite, one reported hypomethylation, while two reported hypermethylation. Five of the epigenome-wide studies showed that the methylation profiles differed between early PE patients and controls, but none or fewer differentially methylated sites were found when comparing late-onset PE and controls [19, 28, 38, 51, 53]. Overall, hypomethylation was the predominant finding in epigenome-wide studies (14/19) analyzing the placental tissue, while hypermethylation was detected in three epigenome-wide studies (3/3) that analyzed maternal WBCs.

**Table 1:** Differentially methylated sites in epigenome-wide studies

Ref. no	Study	Method	Major findings
[17]	Bourque 2010	IlluminaGoldenGateMethylation Cancer Panel 1 array	No significant methylation differences—placenta
[28]	Yuen 2010	IlluminaGoldenGateMethylation Cancer Panel 1	192 loci differentially methylated (hypo) in the placenta in EOPE, none in LOPE.
[30]	Jia 2012	NimbleGen 385K	102 genes in total showed significant hypermethylation in the promoter-associated CpG islands in severe PE placenta tissue samples, while 194 genes showed significant hypomethylation.
[31]	Mousa 2012a	HM 27K	Not reported
[32]	Mousa 2012b	HM 27K	Not reported
[33]	Mousa 2012c	HM 27K	4184 CpG sites (3736 genes) differentially methylated when comparing normal pregnant and preeclamptic omental arteries.
[34]	Blair 2013	HM 450K	38840 CpG sites with significant differences (282 with > 12.5% difference) in EOPE in the placenta. The majority (74.5%) of these sites were hypomethylated in EOPE.
[35]	Hogg 2013a	HM 450K	Not reported
[18]	White 2013	HM 27K	PE was associated with widespread differential methylation favoring hypermethylation in maternal peripheral blood (buffy coat). 729 CpGs were hypermethylated, while 268 were hypomethylated in PE, compared to controls.
[36]	Anderson 2015	HM 450K	Significant differences in DNA methylation were identified in 207 individual CpG sites in WBC, 64% showed a gain, while 36% were associated with a loss of methylation. The majority of the hypermethylated sites in the WBC also showed varying amounts of methylation gain in the placenta tissue, with many sites showing significant methylation gains in the placenta. Also, the hypomethylated sites in WBC were likely to show a loss of methylation to varying degrees in placental tissue, with many sites showing significant methylation losses.
[37]	Anton 2014	HM 450K	3411 gene probes (3132 hypermethylated and 279 hypomethylated) were differentially methylated between control and preterm PE (< 37 gestational weeks) placentas. A total of 179 gene probes (164 hypermethylated and 15 hypomethylated) were differentially methylated between term PE (≥ 37 gestational weeks) and preterm PE placentas.
[38]	Chu 2014	Infinium microarray (EpityperMassARRAY)	49 CpGs significantly altered in EOPE placental tissue compared to normal controls (after excluding X chromosome-specific probes). Seventy-eight percent were hypomethylated in EOPE. Fewer differentially methylated CpGs were also identified when comparing LOPE with controls. Only a single CpG site, MC1R, was found to be differentially methylated between EOPE and LOPE.
[39]	Liu 2014	Methylated-CpG island recovery assay (MIRA)	8191 (2140 genes) differentially methylated regions were identified in PE placentas compared with controls
[40]	Blair 2014	HM 450K	Not reported
[41]	Ching 2014	HM 450K	Of 385,184 useful loci for differential methylation analysis, 9995 showed DM (2.6%) between EOPE and control placentas. 91.9% of those DMs (9186 of 9995) were hypermethylated.
[42]	Anderson 2014	NimbleGen	Not reported
[43]	Ching 2015	HM 450K	Hypomethylation pattern in EOPE in umbilical cord blood with 51,486 hypomethylated CPG sites and 12,563 hypermethylated sites.
[44]	Martin 2015	HM 450K	There were 989 DMPs between the preeclamptic and normotensive placentas. Most (80.7%) of the DMPs were hypomethylated in the preeclamptic placentas versus the normotensive placentas, while only 19.3% were hypermethylated.
[45]	Zhu 2015	[h]MeDIP with MeDIP-seq	A total of 714 differential 5mC peaks (DMRs) were found showing significant differences between late-onset severe PE and controls. Four hundred eighty-seven (68.2%) had higher 5mC levels in the late-onset preeclamptic placentas.
[46]	Xuan 2016	NimbleGen	1664 promoters with altered DNA methylation, including 663 hypermethylated and 1001 hypomethylated in placental samples.
[47]	Kim 2016	HM 450K	Maternal peripheral blood showed 71 differentially methylated CpG loci (44 hypermethylated and 27 hypomethylated), while the placenta revealed 365 loci (37 hypermethylated and 328 hypomethylated).
[48]	Wilson 2016	HM 450K	Not reported
[49]	Suzuki 2016	HELP tagging	123, 85, and 99 loci with high-confidence hypertension-associated, proteinuria-associated, and hypertension- and proteinuria-associated DNA methylation changes in the placenta
[14]	White 2016	HM 27K	Of 73 analyzed CpGs, 6 genes were differentially methylated in PE buffy coat compared to controls
[50]	Yeung 2016	HM 450K	A total of 303 differentially methylated regions after adjustment for gestational age (214 hypermethylated and 89 hypomethylated) between preeclampsia cases and controls in the placenta
[51]	Herzog 2017	HM 450K	5001 mostly hypermethylated DMPs in the umbilical cord (UC)-WBC and 869 mostly hypomethylated DMPs in placental samples. The methylation levels in EOPE UC-WBCs clearly deviated from those in all other groups. In the comparison of EOPE and PTB, we found 12040 (28%) differentially methylated CpGs in UC-WBC and 5668 (0.5%) differentially methylated CpGs in the placenta. One differentially methylated CpG was found in the comparison between EOPE and uncomplicated controls. No epigenome-wide significant CpGs were found in the comparisons of LOPE and (un)complicated controls.
[19]	Van den Berg 2017	HM 450K	DNA methylation significantly differed in EOPE compared with spontaneous preterm birth at 6 CpGs in the placental tissue (hypomethylated), and at 21 CpGs in UC leukocytes (hypermethylated). Moreover, significantly different DNA methylation in EOPE compared with uncomplicated controls was shown at 6 CpGs in the placental tissue and 11 CpGs in uncomplicated controls. No significant associations were shown with LOPE between study groups or tissues. The most differentially methylated CpGs showed hypomethylation in the placental tissue and hypermethylation in UC-WBC.
[52]	Zhao 2017	HM 450K	There were 2667 DMRs (1433 hypermethylated and 1234 hypomethylated) and 464 DMIs between PE and normotensive controls.
[53]	Wilson 2018	HM 450K	1703 sites were differentially methylated in EOPE vs preterm controls, but only a few changes were associated with LOPE compared to term controls in the placenta.
[54]	Wang 2019	HM 450K	A total of 464 probes reached epigenome-wide significance, whilst 459 (98.9%) were hypomethylated in the EOPE placenta in the Chinese cohort.

### Gene-specific DNA methylation—candidate gene studies

For the studies that used candidate-gene approaches, we grouped the findings on DNA methylation and PE according to PE severity. The promoter regions and CpG islands were frequently targeted in these studies. DNA methylation alterations in PE have been analyzed from different biological tissues. The 13 studies demonstrated that the more severe PE cases, compared to controls, had higher degrees of methylation of *H19* [11, 55], *CDH11* [37], *TNF* [37], *LINE-1* [11], *Alu* [11], *NR3C1* [35], *CRHBP* [35], *YWHAQ (T-14-tau)* [56], *DLL1* [57], NuRD [58], BARX [58], MMP9 [59], and VEGF [60] in placental samples, *APC* [61], *HYP2* [62] in plasma, *FAS* [43], *ACTA2* [43], *PI3KR1* [43], *MIR145* [43], *LOC728264* [43], *IL12B* [43], *MIR24-2* [43] in the umbilical cord blood (UCB) cells, and 15 showed lower methylation levels of *NCAM1* [37], *INHBA* [34], *BHLHE40* [34], *SLC2A1* [34], *ADAM12* [34], *TBX15* [43], *LEP* [63], *TERT* [48], *TNFAIP8* [64], *DNMT3A* [48], *VEGF* [60], *AKT1* [19], *CRTC1* [19], *PER1* [19], *CSKN1E* [19], *PRDX1* [19], *RORA* [19], *ARNTL2* [19], *CLOCK* [19], *CRY2* [19], *PER2* [19], *FOXO3* [19], *MAPK1* [19], *PRDX5* [19], *PRKCA* [19], *CAPG* [28], *GLI2* [28], *KRT13* [28], *TIMP-3* [28], E2F4 [58], C12orf7 [54], PVT1_MIR1204 [54], NDUFAF3 [54], ARSG [54], FYCO1 [54], SULF2 [54], PVT1 [54], OBSL1 [54], MERTK [54], C2 [54], PEBP1 [54], SKI [54], CCDC68 [54] in placental samples, APC [65] in serum, *MASPIN* [61] in plasma, and *MMP1* [32] in omental fat arteries (Table 2). One study showed differences in methylation patterns of CpG sites examined at the *DNMT1* [48] (within the same gene cg07627628 was hypomethylated and cg26538782 was hypermethylated). Performing ORA on the genes for severe preeclampsia (including both hyper- and hypo-methylation combined) yielded significant results mostly in the transcription factor binding and regulatory DNA sequence (*p* < 10e−5). No additional specific molecular pathways stood out as a significant after FDR correction.

**Table 2:** DNA methylome alterations in more severe PE cases vs. normotensive controls

Gene	No of CpGs	Region	DNA methylation changes	Sample	PE	Reference
*H19*	/	/	Hypermethylated	Placenta	EOPE	[11]
	7	Exon 1	Hypermethylated	Placenta	sPE	[55]
*CDH11*	1	/	Hypermethylated	Placenta	EOPE	[37]
*NCAM1*	1	/	Hypomethylated	Placenta	EOPE	[37]
*TNF*	1	/	Hypermethylated	Placenta	EOPE	[37]
*INHBA*	1	5′ UTR	Hypomethylated	Placenta	EOPE	[34]
*BHLHE40*	1	Body	Hypomethylated	Placenta	EOPE	[34]
*SLC2A1*	1	Body	Hypomethylated	Placenta	EOPE	[34]
*ADAM12*	1	Body	Hypomethylated	Placenta	EOPE	[34]
*LINE-1*	/	/	Hypermethylated	Placenta	EOPE	[11]
*TBX15*	39	Promoter	Hypomethylated	Placenta	EOPE	[43]
*Alu*	/	/	Hypermethylated	Placenta	EOPE	[11]
*NR3C1*	Multiple	promoter	Hypermethylated	Placenta	EOPE	[35]
*CRH*	Multiple	5′ UTR	No difference	Placenta	EOPE	[35]
*CRHBP*	Multiple	Intron 3	Hypermethylated	Placenta	EOPE	[35]
*LEP*	Multiple	Promoter	Hypomethylated	Placenta	EOPE	[63]
*TERT*	1 (cg01934390)	/	Hypomethylated	Placenta	EOPE	[48]
*TNFAIP8*	1	/	Hypomethylated	Placenta	EOPE	[64]
*YWHAQ (T-14-tau))*	19	Promoter	Hypermethylated	Placenta	sPE	[56]
*WNT2*	7	Promoter	No difference	Placenta	EOPE	[66]
*DNMT1*	1 (cg07627628)	/	Hypomethylated	Placenta	EOPE	[48]
	1 (cg26538782)	/	Hypermethylated			
*DNMT3A*	1 (cg11779362)	/	Hypomethylated	Placenta	EOPE	[48]
*DLL1*	/	Promoter	Hypermethylated	Placenta	EOPE	[57]
*NOTCH1*	/	Promoter	No difference	Placenta	EOPE	[57]
*VEGF*	23	Promoter	Hypermethylated	Placenta	EOPE	[60]
*FLT-1*	30	Promoter	No difference	Placenta	EOPE	[60]
*KDR*	37	Promoter	No difference	Placenta	EOPE	[60]
*AKT1*	1	/	Hypomethylated	Placenta	EOPE	[19]
*CRTC1*	1	/	Hypomethylated	Placenta	EOPE	[19]
*PER1*	1	/	Hypomethylated	Placenta	EOPE	[19]
*CSKN1E*	1	/	Hypomethylated	Placenta	EOPE	[19]
*PRDX1*	1	/	Hypomethylated	Placenta	EOPE	[19]
*RORA*	1	/	Hypomethylated	Placenta	EOPE	[19]
*ARNTL2*	1	/	Hypomethylated	Placenta	EOPE	[19]
*CLOCK*	2	/	Hypomethylated	Placenta	EOPE	[19]
*CRY2*	3	/	Hypomethylated	Placenta	EOPE	[19]
*PER2*	4	/	Hypomethylated	Placenta	EOPE	[19]
*FOXO3*	5	/	Hypomethylated	Placenta	EOPE	[19]
*MAPK1*	2	/	Hypomethylated	Placenta	EOPE	[19]
*PRDX5*	1	/	Hypomethylated	Placenta	EOPE	[19]
*PRKCA*	2	/	Hypomethylated	Placenta	EOPE	[19]
*CAPG*	2	Promoter	Hypomethylated	Placenta	EOPE	[28]
*GLI2*	2	Promoter	Hypomethylated	Placenta	EOPE	[28]
*KRT13*	2	Promoter	Hypomethylated	Placenta	EOPE	[28]
*NuRD*	/	Promoter	Hypermethylation	Placenta	EOPE	[58]
*BARX*	/	Promoter	Hypermethylation	Placenta	EOPE	[58]
*E2F4*	/	Promoter	Hypomethylation	Placenta	EOPE	[58]
*MMP9*	1	Promoter	Hypermethylation	Placenta	sPE	[59]
*C12orf75*	1	body	Hypomethylation	Placenta	EOPE	[54]
*PVT1_MIR1204*	1	TSS200	Hypomethylation	Placenta	EOPE	[54]
*NDUFAF3*	1	TSS200	Hypomethylation	Placenta	EOPE	[54]
*ARSG*	1	body	Hypomethylation	Placenta	EOPE	[54]
*FYCO1*	1	body	Hypomethylation	Placenta	EOPE	[54]
*SULF2*	1	5′ UTR	Hypomethylation	Placenta	EOPE	[54]
*PVT1*	1	body	Hypomethylation	Placenta	EOPE	[54]
*OBSL1*	1	body	Hypomethylation	Placenta	EOPE	[54]
*MERTK*	1	body	Hypomethylation	Placenta	EOPE	[54]
*C2*	1	1st exon	Hypomethylation	Placenta	EOPE	[54]
*PEBP1*	1	body	Hypomethylation	Placenta	EOPE	[54]
*SKI*	1	body	Hypomethylation	Placenta	EOPE	[54]
*CCDC68*	1	TSS200	Hypomethylation	Placenta	EOPE	[54]
*DKK1*	5	Promoter	No difference	Placenta	EOPE	[66]
*TIMP-3*	/	/	No difference	Serum	sPE	[65]
	2	Promoter	Hypomethylated	Placenta	EOPE	[28]
*RASSF1A*	/	/	No difference	Serum	sPE	[65]
*CDH1*	/	/	No difference	Serum	sPE	[65]
*PTGS2*	/	/	No difference	Serum	sPE	[65]
*BLT1*	/	/	No difference	Serum	sPE	[65]
*APC*	/	/	Hypomethylated	Serum	sPE	[65]
	/	/	Hypermethylated	Plasma	sPE	[61]
*HYP2*	/	/	Hypermethylated	Plasma	EOPE	[62]
*MASPIN (SERPINB5)*	/	/	Hypomethylated	Plasma	sPE	[61]
*FAS*	1	/	Hypermethylated	UCB	EOPE	[43]
*ACTA2*	1	/	Hypermethylated	UCB	EOPE	[43]
*PI3KR1*	1	/	Hypermethylated	UCB	EOPE	[43]
*MIR145*	2	/	Hypermethylated	UCB	EOPE	[43]
*LOC728264*	2	/	Hypermethylated	UCB	EOPE	[43]
*IL12B*	1	/	Hypermethylated	UCB	EOPE	[43]
*IGF1*	1	/	No difference	UCB	EOPE	[43]
*MIR24-2*	1	/	Hypermethylated	UCB	EOPE	[43]
*MMP1*	1	Promoter	Hypomethylated	Omental fat arteries	sPE	[43]

Two studies found higher levels of methylation of *ACAP2* [45], *CLIC6* [45], *GATA4* [45], *PCDH9* [45], *CCDC149* [45], *PTPRN2-A* [45], *PTPRN2-B* [45], and *RBFOX1* [45], and lower levels of methylation of *INHBA* [67], *BHLHE40* [34], *SLC2A1* [34], and *PTPRN2-A* [45] in the plasma tissue of less severe PE patients compared to normotensive controls (Table 3). For studies without data on PE severity, the list of differentially methylated genes is given in Additional file 1: Table S5. In candidate genes studies, a higher number of differentially methylated genes was found when analyzing more severe PE (70/82), compared to studies analyzing less severe PE vs. controls (13/27).

**Table 3:** DNA methylome alterations in less severe PE cases vs. normotensive controls

Gene	No of CpGs	Region	DNA methylation changes	Sample	PE	Reference
*COL5A1*	1	/	No difference	Placenta	LOPE	[37]
*INHBA*	2	5′ UTR	Hypomethylated	Placenta	LOPE	[34]
*BHLHE40*	1	Body	Hypomethylated	Placenta	LOPE	[34]
*SLC2A1*	1	Body	Hypomethylated	Placenta	LOPE	[34]
*ADAM12*	1	Body	No difference	Placenta	LOPE	[34]
*LINE-1*	/	/	No difference	Placenta	LOPE	[11]
*Alu*	/	/	No difference	Placenta	LOPE	[11]
*CRH*	Multiple	5′ UTR	No difference	Placenta	LOPE	[35]
*CRHBP*	Multiple	Intron 3	No difference	Placenta	LOPE	[35]
*LEP*	Multiple	Promoter	No difference	Placenta	LOPE	[63]
*TERT*	1 (cg01934390)	Promoter	No difference	Placenta		[48]
	1 (cg11832804)	Promoter			LOPE	
*DNMT1*	1 (cg07627628)	/	No difference	Placenta		[48]
	1 (cg26538782)	/			LOPE	
*NOTCH1*	/	Promoter	No difference	Placenta	LOPE	[57]
*VEGF*	23	Promoter	Hypomethylated	Placenta	LOPE	[60]
*FLT-1*	30	Promoter	No difference	Placenta	LOPE	[60]
*KDR*	37	Promoter	No difference	Placenta	LOPE	[60]
*ACAP2*	4	Promoter	Hypermethylated	Placenta	LOPE	[45]
*CLIC6*	11	Promoter	Hypermethylated	Placenta	LOPE	[45]
*GATA4*	9	Promoter	Hypermethylated	Placenta	LOPE	[45]
*PCDH9*	7	Promoter	Hypermethylated	Placenta	LOPE	[45]
*PTPRN2-A*	20	Promoter	Hypomethylated	Placenta	LOPE	[45]
*CCDC149*	/	Promoter	Hypermethylated	Placenta	LOPE	[45]
*PTPRN2-A*	/	Promoter	Hypermethylated	Placenta	LOPE	[45]
*PTPRN2-B*	/	Promoter	Hypermethylated	Placenta	LOPE	[45]
*RBFOX1*	/	Promoter	Hypermethylated	Placenta	LOPE	[45]
*MASPIN (SERPINB5)*	/	/	No difference	Plasma	mPE	[61]
*MMP1*	1	Promoter	No difference	Omental fat arteries	mPE	[32]

### Methodological study characteristics

We evaluated methodological study characteristics, including design (number of participants, study design, and PE severity), implementation (biological sample, DNA methylation method, purification of DNA extraction, and validation of methylation), analysis (analytical method, batch effect, genotyping, and gene expression), data presentation (methylation quantification measure, measures of variability, reporting), and major epigenetic findings. Ninety eligible articles were identified, including a total of 6197 participants (2536 PE vs. 3661 controls). Preeclampsia severity was reported in 35 studies. Women with less severe PE were included in 18 studies, while women with more severe PE were included in all 35 studies. There were eighteen case-control studies, with a total of 2219 cases and 2671 controls. The most frequent study design was cross-sectional (*n* = 62). The remaining studies were nested case-control (five studies), and four prospective cohorts which included 107 participants. Participants were matched in 30% of studies, most often by gestational age (15/27) (Additional file 1: Table S1). In only six studies, participants with PE and those in the control group were matched by maternal age (Additional file 1: Table S1). In four studies, an analytical approach (ANCOVA, regression analysis) was applied to control for confounding variables. Women’s ethnicities were reported in 18 studies. Homogeneous ethnic groups were identified in three studies, and adjustment for this factor was applied in five studies. Fetal gender was considered as an important factor influencing the DNA methylome in 15 studies [13, 28, 29, 34, 38, 40, 41, 43, 48–50, 53, 63, 68, 69]. In nine studies, all probes on the X chromosome were excluded; in two studies, the genders were analyzed separately, and in five studies, adjusting for fetal gender was applied.

Table 4 summarizes the implementation characteristics of the studies included in this review. The most commonly applied time of sampling in both groups was at delivery, which was reported in 82 (91%) studies, except for seven of these studies in which the time of sampling in the control group was reported as the time of abortion, including abortions in the 1st and 2nd trimesters of pregnancy. The frequency of sampling before delivery was 14%, while five studies reported sampling after delivery. The time of sampling was not reported in seven of the studies. Placental samples were the most common studied tissues, as they were used for DNA methylome profiling in 75 studies (83%). Of 25 studies in which the maternal peripheral blood was sampled, plasma samples were used in nine studies, serum samples in two studies, WBC in nine studies, and lymphocytes in only one study. In a small number of studies, DNA methylation was assessed from the umbilical cord blood cells or omental fat arteries (seven and three studies, respectively). Two studies used the vessels as samples (one placental vessel and one umbilical vein). Fifty-eight studies used the candidate-gene approach and 34 examined the whole genome. Sequencing-based methods were widely used to quantify levels of DNA methylation across all studies. The most frequently reported was bisulfite sequencing (25%), followed by bisulfite pyrosequencing, which was used in 21% of studies. Genome-wide microarray technologies, such as Illumina27, Illumina450, or Illumina HiSeq2500, were used in 23% of the reviewed studies. All other methods were reported in a small number of studies. Almost 40% of these studies checked DNA purification (33/90), but there were fewer that included methylation validation (30/90).

**Table 4:** Implementation characteristics of studies

Characteristics	***n***	%
**Time of sampling**		
At the time of delivery	82	91.1
Before delivery	13	14.4
After delivery	5	5.5
At the time of abortion	7	7.8
Not reported	7	7.8
**Sample**		
Placenta	75	83.3
Maternal peripheral blood		
Plasma	9	10.0
Serum	2	2.2
White blood cells (WBC)	9	10.0
Lymphocytes	1	1.1
Whole maternal blood	1	1.1
Umbilical cord blood	7	7.8
Omental fat arteries	3	3.3
Placental vessels	1	1.1
Umbilical vein	1	1.1
**Targeted genetic locations**		
Global DNA-methylation	5	5.5
Genome-wide	4	4.5
Genome-wide, selected genes for replication	25	27.8
Candidate gene(s)	58	64.4
**DNA methylation method (most frequent)**		
Illumina27+Illumina450	21	23.3
Bisulfite sequencing	23	25.5
Bisulfite pyrosequencing	19	21.1
Methylation specific PCR	11	12.2
**Purification of DNA extraction**	33	36.7
**Validation of methylation**	30	33.3

Data were analyzed using both parametric and non-parametric tests. Batch-effect correction was found in 9 studies only. Only seven studies performed genotyping, while more than half (61/90) conducted gene-expression studies.

A high degree of heterogeneity existed among the studies in terms of data presentation (Table 5). In more than one-half of these studies, the mean DNA methylation level was reported using a percentage value. *β* values were found in 19 studies, and Δ*β* values were reported in 15 of the reviewed studies. One-half of the studies (56%) reported a measure of variability (standard deviation–sd, standard error—se, 95% confidence interval—95%CI, interquartile range—iqr, range) for the level of DNA methylation in each group. Also, individual data were found in a small number of studies (14%). Values for the DNA methylation level were commonly graphically presented (78%), and only one third of the reviewed studies reported mean levels of DNA methylation in their tables. Most importantly, we found row data stored in a Gene Expression Omnibus repository for only eleven of the studies included in this review [18, 34, 35, 37, 40, 44, 49–51, 53, 70].

**Table 5:** Data presentation

Data presentation	***n***	%
**Methylation quantified by**		
*β* value	19	21.1
Δ*β*	15	16.7
Adjusted Δ*β*	1	1.1
Δ*β* fold change	1	1.1
Fold change	4	4.5
Relative fold change	1	1.1
Log_2_ ratio	7	7.8
Log_2_ fold change	1	1.1
%^*^	71	78.9
*M* value (logit transformed *β*)	3	3.3
Diff score	1	1.1
Ct (ΔCt, 2- ΔCt)	2	2.2
H-score	1	1.1
Positive for methylation	1	1.1
Number of copies/mL	2	2.2
**Error presented** (sd, se, iqr, 95%CI, range)	50	55.5
**Individual data**	13	14.4
**Methylation reported in**		
Main text		
Table	36	40.0
Figure	70	77.8
In text only	2	2.2
Supplement	17	18.9
Not reported at all	1	1.1
**Repository**	11	12.2

*β* value is an estimate of methylation level using the ratio of intensities between methylated and unmethylated alleles *β* = methylated allele intensity (M)/(unmethylated allele intensity (U) + methylated allele intensity (M) + 100); ^*^%, mean methylation %, % change in methylation, % of methylated/unmethylated, % hypermethylated; using the Illumina Custom Algorithm a Diff score is calculated from the *p* value of significance: DiffScore = (10sgn(Icond-Iref)log_10_(p)); Ct value is a relative measure of DNA methylation level that denotes which cycle the fluorescence goes over a certain threshold value; H-score is the product of the percentage of cells in each sample with positive staining (range, 0–100%) multiplied by the intensity of staining (range, 0–3); sd, standard deviation; se, standard error; iqr, interquartile range

## Discussion

This systematic review aimed to summarize the findings on the association between DNA methylation and preeclampsia and to explore the major study characteristics that can significantly impact this association. Overall, altered methylation (hyper- and hypo-) on the promoter regions and CpG islands of a number of genes were found in both more and less severe forms of PE, and hundreds of DMPs/DMRs were identified in the studies that examined the whole genome. A high degree of heterogeneity existed among the studies in terms of methodological study characteristics.

### Association between DNA methylation and preeclampsia

Global methylation levels were evaluated in different tissues with inconsistent conclusions. Placental global hypomethylation in normal pregnancy changes throughout gestation [71, 72]. LINE-1 (Long Interspersed Nucleotide Element 1) serves as a surrogate for global DNA methylation levels [73]. This systematic review showed that LINE-1 was hypermethylated in placentas from early-onset PE patients, without significant difference in late-onset PE, compared to healthy pregnant women’s placentas. This finding could explain the fluctuation of DNA methylation levels during pregnancy trimesters and also the differences in PE phenotypes. In that context, Myatt and coworkers suggested the use of preterm deliveries as an adequate control group for early-onset PE [74].

EWAS found significant methylation sites, including hyper- and hypomethylation. The majority of the studies analyzed DNA methylation levels in the placenta, and 73% of them reported significant hypomethylation sites. In addition, the methylation profiles for EOPE and LOPE were reported to be different, thus suggesting distinct phenotypes [75]. However, the observed methylation difference may be due to differences in gestational age between these two forms of PE. The availability of control placentas throughout gestation facilitates making distinctions between the effects of gestational age and those related to PE subtype. Additional limitations of published studies include the cellular complexity of the examined tissues, with more than one cell type commonly being present in the examined samples, and the various statistical tests that were utilized. Furthermore, studies rarely included replication cohorts. Konwar et al. suggested that addressing the abovementioned aspects would facilitate the reproducibility and integration of results across placenta studies when processing and interpreting genomics data [76].

While DNA methylation changes of candidate genes in placental and other tissues have been confirmed by EWAS, only a few new loci of interest have been discovered [34, 37, 59]. Blair et al. reported four genes relevant to preeclampsia (INHBA, BHLHE40, SLC2A1, and ADAM12), with different degrees of methylation changes in LOPE compared to EOPE [34]. Previous studies have shown that BHLHE40 [77] and SLC2A1 [78] expressions can be affected by hypoxia, while INHBA and ADAM12 [79] are relevant to prenatal screening. One study [35] showed increased DNA methylation at CpG sites within genes encoding the glucocorticoid receptor (NR3C1) and CRH binding protein (CRHBP) in EOPE-associated placentas, but not in LOPE compared to controls. In the same study, significant hypomethylation was observed in EOPE, but not in LOPE placentas for the TEA domain family member 3 (TEAD3) and CYP19 [35]. The same authors in another publication found that the DNA methylome level of the LEP gene promoter was hypomethylated in EOPE, but not in LOPE [63]. Robinson et al. reported that after adjusting for fetal sex and gestational age, DNA methylation at cg01934390 within TERT and cg26538782 within DNMT1 differed between EOPE and controls, and no difference was observed between LOPE and controls [48]. The results of Sundrani et al. showed a significantly lower mean methylation level of the vascular endothelial growth factor (VEGF) promoter, an important mediator in many endothelial and extravascular processes, in the preterm PE group compared to the normotensive group [60, 80]. They showed significantly reduced mean methylation at the CpG site 6, 7, and CpG site 8, and significantly higher methylation at CpG site 14 [60]. Also, the mean methylation at CpG site 16 in the FLT-1 (VEGFR1—vascular endothelial growth factor receptor) promoter region was significantly reduced in the term PE group compared to the normotensive group, while mean methylation at CpG site 17 was significantly reduced in the preterm PE group compared to the normotensive group [60]. Qi et al. found that the u-maspin (or SERPINB5 gene) ΔCt value was significantly lower in women with severe PE compared to those with a normal 3rd-trimester pregnancy, and no difference was found between women with mild PE and those with a normal 3rd-trimester pregnancy [61]. Mousa et al. reported a significant decrease in methylation in the promoter region of the MMP1 gene in the omental arteries of women with severe PE compared to normal pregnant women, but methylation at the same site was not significantly different in the arteries obtained from women with mild PE compared to the arteries from normal pregnant women [32]. Although methylations are found mostly in the regulatory (promoter and enhancer) regions of genes, the fact that the genes themselves are transcription factors indicates that preeclampsia is a multi-level regulatory disturbance with widespread genomic effects rather than affecting a singular pathway.

### Study characteristics

The basic necessary step for the realization of quality research is defining a clear understandable hypothesis referring to the specific disease phenotype [81]. Clear conclusions are expected to be found in a more specific form of the disease (more or less severe preeclampsia), because of the differences in etiology, pathophysiology, course, and also the prognosis of the disease [82]. A good hypothesis should be followed by an adequate epidemiological study design. The majority of reviewed studies were retrospective, while a more appropriate, nested case-control with a prospective cohort design [83] was used in just nine studies. The major reason for recommending a prospective study design is to avoid a list of potential confounders [83, 84]. Many environmental (chemical exposure, nutrition) and lifestyle characteristics (smoking, alcohol, and drug consumption) [85, 86] can influence the strength or even change the direction of associations between DNA methylation (global or site specific) and preeclampsia. The most important factor, gestational age, strongly influences the DNA methylation level in placental samples [87]. DNA methylation level is also related to maternal age [88] and the risk for developing PE [78, 89]. However, only six studies were participants matched for maternal age or an adequate method employed to control for maternal age. Fetal gender also influences DNA methylation level, and previous findings have shown that there is a sexual epigenetic dimorphism of the placental DNA methylome [90]. Tissue samples from pregnancies with a female fetus have a larger number of differentially methylated loci [32], suggesting the importance of considering fetal gender when assessing the DNA methylation level in pregnant women.

Most of the included studies had disadvantages in terms of scarcity of patient characteristics, preeclampsia definitions, and reporting of inclusion and exclusion criteria. Comprehensive data on patient characteristics as potential confounders are of great importance for obtaining accurate conclusions. The variety of reported PE definitions found in the reviewed studies does not allow for generalization of the conclusions. Although more (early/severe) and less (late/mild) severe PE may have different epigenetic pathophysiologies, disease severity was reported in fewer than half of the studies. Complete or incomplete data on pre-existing hypertension, previous history of PE, positive family history for hypertensive disorders in pregnancy, other cardiovascular diseases, renal diseases, and diabetes mellitus or additional inclusion or exclusion criteria were reported in 66 (73%).

The tissue specificity of the epigenome pattern adds another challenge to the design of DNA methylation studies in preeclampsia. DNA methylation level was analyzed predominantly in the placental tissue (83.3%) rather than in extra-embryonic membranes (amniotic and chorionic). The placenta is a dynamically changing organ. Fine-tuning sensitive mechanisms such as angiogenesis and the fetal–maternal interface and adequate cytotrophoblast invasion with the remodeling of the spiral arteries are crucial for physiological placentation. Adverse pregnancy outcomes, such as preeclampsia, could be the results of these processes [91]. In an effort to improve reporting standards in studies of human placenta, the use of a consistent sampling location and focusing on a specific cell type have been recommended [84]. Maternal peripheral blood was sampled in 27.8% of studies. Plasma was the most commonly analyzed of all blood derivatives (in 36%). Specific types of blood cells were rarely used for DNA methylation analysis in PE. Purified samples consisting only of a single-cell type have been recommended instead of mixed cell samples [81]. To date, limited or no work has addressed the stability and correlation of DNA methylation patterns in different blood products, as they relate to preeclampsia. Differences in DNA methylation levels between PE and normotensive women [18], as well as changes in DNA methylation levels during normal and pathologic pregnancies within the same tissue type [14, 92] were reported. The importance of sample specificity is further reflected in the presence of proven differences in methylation levels within different tissue types in preeclampsia, such as hypermethylation in umbilical cord-WBCs and hypomethylation in the placental tissue in EOPE vs. preterm normotensive controls [51]. Because cell specificity has been ignored in most methylation studies, it is difficult to interpret results and draw meaningful conclusions. Tissue specificity of epigenetic patterns and heterogeneity in sample collection are just some of the obstacles that have hindered progress in identifying mechanisms involved in the pathogenesis of PE and identifying relevant clinical applications.

DNA methylation as a potential mechanism of transcription and gene expression changes could be evaluated in blood cellular components during pregnancy, at the time of a PE diagnosis or after delivery. The time of sampling is indicated by the type of the sampled tissue. The timing to obtain the clinically most relevant result would theoretically be before diagnosis, as early as possible during pregnancy. Scientific relevance, on the other hand, can be demonstrated regardless of the timing of tissue sampling, in order to explore the changing of DNA methylation levels involved in PE pathogenesis.

The methodology used for methylation analysis varied among the studies. Mostly, well-established techniques such as bisulfite polymerase chain reaction (PCR) and pyrosequencing or 27 K or 450 K BeadChip arrays were performed. Choosing an adequate technique depends on the aim of the study, the amount and quality of the DNA sample, the sensitivity and specificity required in the study, and the needed robustness of the method, as well as availability of software, equipment, reagents, and also costs. For many years, bisulfite sequencing PCR (BSP) has been the “gold standard” for measuring DNA methylation, although this method is not able to distinguish methylcytosine (5-mC) from cytosine [73]. The ability of pyrosequencing to reliably detect differences in DNA methylation, using previously designed primers, across cell populations without requiring the cloning of bisulfite-treated DNA, is a major advantage of this technique [93, 94]. It is a good technique for heterogeneous samples, where only a fraction of cells has a differentially methylated gene of interest [73]. Because of its time-saving and cost-efficiency, ability to analyze many identified sites, and also quantitative accuracy and reproducibility, HM450 has become the most widely used epigenotype-mapping tool. Cross-reactive probes, SNP-affected probes, within-array bias (Infinium I and II bias), and between-array bias (batch effects) are important weaknesses inherent with the use of HM450 epigenotype mapping, especially when subtle methylation differences need to be detected by statistical tests between large numbers of cases and controls [95, 96]. Recently, newly introduced sophisticated methods, such as next-generation sequencing (NGS), present a huge potential for future research [97].

More than half of the reviewed studies used a candidate gene approach. DNA purification and DNA methylation validation of findings across multiple independent samples or cohorts are crucial for accurate results. Purification of extracted DNA was performed in fewer than half of the studies. A small number of studies performed validation, in the same or a separate study population. Although batch effects (differences between study groups caused by the heterogeneity of laboratory conditions, reagents, and respondent characteristics) may bias methylation study results, they were corrected in only 9 studies. Not controlling for batch effects remains a significant problem that leads to incorrect conclusions. It is recommended that SNP (single-nucleotide polymorphism) genotyping be performed in order to obtain more precise conclusions. This process is not only important for GWAS studies, but also for epigenome studies [98]. It can help in finding a specific genotype associated with a specific methylation level change in PE. Only half of the reviewed studies analyzed correlations between methylation alterations and gene expression. As gene-expression determines whether the association between DNA methylation and PE represents a true biological effect, future methylation studies should assess gene expression.

There was an effort to standardize methodology reporting after 2011, but it has not been sustained. Purification of extracted DNA and gene-expression have often been reported in articles published during the last few years, but data for genotyping and controlling for batch effects are still insufficient. The deposition of data in repositories began in 2013, but it is still a rare practice, being found in only a small number of the included studies.

Improved and standardized reporting of DNA methylation should be necessary for the identification of epigenetic-based effects in preeclampsia. Methylation levels were commonly reported using percentage values and *β* values as recommended [73], but often in figures only and without an accompanying measure of variability. A small number of studies included individual data and deposited raw data in open repositories. It is recommended that DNA methylation results should be presented in tables using the arithmetic mean with standard deviation or median with range or an interquartile range. In view of new attitudes towards better data reproducibility, it is also recommended that raw data should be saved in on-line repositories [84].

The findings of this review support the role of DNA methylation in the pathophysiology of preeclampsia. In candidate gene studies, methylation alterations for a number of genes were found to be associated with PE. EWAS identified hundreds of DMPs/DMRs in preeclamptic patients, further supporting the abnormality of the DNA methylome in PE. However, methodological problems found in the reviewed studies make it difficult to draw definitive conclusions regarding the association between DNA methylation and preeclampsia. Establishing field-wide methodological and analytical standards for rigorous and reproducible study designs, sample processing and data analyses may increase value and reduce waste, allowing researchers to gain additional insights into the role of DNA methylation in the pathophysiology of preeclampsia. As a result of the current review, we suggest that future epigenetic studies of methylation in preeclampsia consider the following recommendations:

- Utilize a prospective (cohort) or conceivably a nested case control within a cohort rather than a retrospective study design
- Define preeclampsia according to the recommended guidelines and take into account disease severity, i.e., EOPE, LOPE, mPE, and sPE
- When defining the control group, take into account gestational age, the health status of the target population (healthy or without hypertensive pregnancy disorders/PE), inclusion/exclusion of women with chronic hypertension
- Account for confounders, i.e., maternal age, gestational age, parity, ethnicity, fetal gender, comorbidities, environmental (chemical exposure, nutrition), and lifestyle characteristics (smoking, alcohol, and drug consumption)
- Choose an appropriate tissue and control for cellular heterogeneity, i.e., choose a tissue that allows for individual cell counts and/or computational correction. Analyze changes in methylation level over time, i.e., maternal peripheral blood in the 1st, 2nd or 3rd trimester, at the time of delivery, after delivery
- Report details about the purification of the extracted DNA molecule
- Epigenome-wide approaches using a bead chip may be useful for hypothesis generation (larger sample sizes likely required)
- Targeted bisulfite sequencing may be useful to capture DNA methylation in a focused region for a specific hypothesis-driven approach addressing a specific biological question, thus allowing for comprehensive methylation analyses of a particular site
- Include a validation cohort, preferably from a different population
- Plan to analyze gene expression to evaluate the effect of methylation
- Choose an adequate methylation quantification measure and report exact values with the appropriate measure of variability, i.e., arithmetic mean with standard deviation, median with range or interquartile range
- Report details as to the applied specific analytical methods
- Be transparent in data presentation and store row data in open repositories
- Minimize unseen variation by reporting all the relevant metadata and labeling technical batches

### Perspectives and significance

Increased collaboration within the scientific community aiming to establish field-wide methodological and analytical standards may allow additional insights into the role of DNA methylation in the pathophysiology of preeclampsia. Sharing of public datasets, with the inclusion of sampling protocols, relevant technical variables, such as DNA quality assessment, batch/chip design maps, pre-processed data, and confounders, may provide sufficient sample sizes, allowing for definitive conclusions about the association between DNA methylation and preeclampsia.

## Supplementary information

**Additional file 1: Table S1.** Summary of Studies included in the systematic review; **Table S2.** Definitions of preeclampsia; **Table S3.** Criteria for less severe and more severe forms of preeclampsia; **Table S4.** Exclusion criteria for studies examining DNA methylation between preeclamptic and normotensive women; **Table S5.** Differentially methylated genes in not specified PE.

## Data Availability

All data generated or analyzed during this study are included in this published article [and its supplementary information files].

## Abbreviations

PE: Preeclampsia
EOPE: Early-onset preeclampsia
LOPE: Late-onset preeclampsia
DMPs: Differentially methylated probes
DMRs: Differentially methylated regions
sPE: Severe preeclampsia
mPE: Mild preeclampsia
