# Supplementary material for: Systematic review supports the role of DNA methylation in the pathophysiology of preeclampsia: a call for analytical and methodological standardization
Source: Biol Sex Differ. 2020 Jul 6;11:36. doi: 10.1186/s13293-020-00313-8 (PMC7336649; doi:10.1186/s13293-020-00313-8)
Supplement: Supplementary file 1 — Additional file 1: Table S1. Summary of Studies included in the systematic review; Table S2. Definitions of preeclampsia; Table S3. Criteria for less severe and more severe forms of preeclampsia; Table S4. Exclusion criteria for studies examining DNA methylation between preeclamptic and normotensive women; Table S5. Differentially methylated genes in not specified PE. [file 13293_2020_313_MOESM1_ESM.docx]

**Table of content: page**

Table S1: Summary of studies included in the systematic review 2

Table S2. Definitions of preeclampsia 13

Table S3. Criteria for less severe and more severe forms of preeclampsia 17

Table S4. Exclusion criteria for studies examining DNA methylation between preeclamptic and normotensive women 20

Table S5. Differentially methylated genes in not specified PE 27

Table S1: Summary of studies included in the systematic review

| No | Study  Country | Study design | Sample size |  | Age in PE vs. Controls | Sample | Time of sampling | Controls/Unexposed | Matching | Inclusion criteria | | |
| --- | --- | --- | --- | --- | --- | --- | --- | --- | --- | --- | --- | --- |
|  |  |  | PE | Controls/ Unexposed |  |  |  |  |  | All Primiparas | All Non-smokers | No Chronic Hypertension |
| (1) | Muller 2004  Austria | case-control | 17(sPE+E+HELLP) | 32 | 32 (19.7-41.3) vs  28.5 (8.6-42.4) | maternal peripheral blood (serum) | early gestational weeks | healthy pregnant women with normal pregnancy outcomes | no | Not reported | Not reported | unclear |
| (2) | Chelbi 2007  France | cross-sectional | 47 | 18 | 32.3±7.7  vs.  36.7±5.4 | placenta | at the time of delivery | women who underwent Cesarean surgery without suffering any disease during pregnancy | no | not reported | not reported | not reported |
| (3) | Tsui 2007  China | cross-sectional | 10 | 20 | Not reported | placenta | at the time of delivery | normotensive pregnant women | Gestational age | Not reported | Not reported | + |
| (4) | Bellido 2010  Switzerland | cross-sectional | 8 | 25 | Not reported | placenta | at the time of delivery | pregnant women without PE | no | not reported | not reported | not reported |
| (5) | Bourque 2010^*^  Canada | cohort | 17 | 22 | Not reported | placenta | at the time of delivery | women who did not develop PE | no | not reported | not reported | unclear |
| (6) | Wang 2010  China | cross-sectional | 20 | 18 (PE+IUGR) | 28.4±3.2  vs.  28.4±4.3 | placenta | at the time of delivery | pregnant women who underwent Cesarean section without suffering from any disease during pregnancy | no | Not reported | Not reported | + |
| (7) | Yuen 2010  Canada | cross-sectional | 8  (EOPE 4, LOPE 4) | 9  (early 4, late 5) | 33.3 (EOPE), 36.45 (LOPE)  vs.  36.03 (early controls), 37.16 (late controls) | placenta | at the time of delivery | gestation matched pregnant women without PE | Gestational age | Not reported | Not reported | + |
| (8) | Zhao 2010  China | cross-sectional | 20 | 60 | 31.1±6.5  vs.  30.4±4.2 | maternal peripheral blood (plasma) | on admission and 24 hours and 7 days after delivery | 1st, 2nd and 3rd trimester normal pregnancy | no | Not reported | Not reported | Not reported |
| (9) | Chelbi 2011  France | cross-sectional | 16 | 15 | 33.60±6.07  vs.  34.40±4.88 | placenta | at the time of delivery | women who underwent Cesarean surgery without suffering any disease during pregnancy | no | not reported | + | + |
| (10) | Gao 2011  China | nested case-control | 24  (EOPE 10, LOPE 14) | 24 | 31.20±5.14 EOPE, 30.36±3.67 LOPE  vs.  30.56±4.08 | placenta | at the time of delivery | normotensive women without PE | no | not reported | not reported | + |
| (11) | Kulkarni 2011  India | cross-sectional | 57 (term 30, preterm 27) | 30 | 22.27±2.97 term, 23.96±3.68 preterm  vs.  22.87±3.16 | placenta | at the time of delivery | normotensive women in singleton pregnancy | no | not reported | + | + |
| (12) | Zhao 2011  China | cross-sectional | placenta 16maternal peripheral blood 4umbilical cord blood 8 | placenta 21maternal peripheral blood 6umbilical cord blood 8 | Not reported | placenta  maternal peripheral blood  umbilical cord blood | at the time of delivery  immediately prior to the obstetrical procedures  at the time of delivery | normal pregnant women | Gestational age | Not reported | Not reported | + |
| (13) | Jia 2012  China | cross-sectional | 9 | 9 | 29.0±2.9  vs.  28.0±2.6 | placenta | at the time of delivery | pregnant women without PE | no | not reported | not reported | + |
| (14) | Mousa 2012a  USA | cross-sectional | 7 | 5 | 24.9±1.4  vs.  23.0±1.7 | omental fat arteries | at the time of delivery | normotensive pregnant women | no | - | + | + |
| (15) | Mousa 2012b  USA | cross-sectional | 21 | 18 | 24.6±1.0  vs.  26.1±1.3 | omental and subcutaneus fat arteries | at the time of delivery | normotensive pregnant women | no | - | + | + |
| (16) | Mousa 2012c  USA | cross-sectional | 7 | 5 | 25.6±4.9  vs.  26.8±6.2 | omental fat arteries | at the time of delivery | normotensive pregnant women | no | - | + | + |
| (17) | Blair 2013  Canada | case-control | 20 | 20 | 31.8 (24.9-37.3)  vs.  31.8 (25.0-37.3) | placenta | at the time of delivery | chromosomally normal losses or births due to a mix of etiologies (premature rupture of membranes, loss of amniotic fluid and cervical incompetence) with no evidence of placental abnormality based on a pathological exam | Gestational age | not reported | not reported | + |
| (18) | Czikk 2013  Canada | cross-sectional | 7 | 9 | 31.7±6.7  vs.  31.1±5.4 | placenta | first and early second trimester villous tissues from elective social terminations of pregnancy  at the time of delivery (term pregnancies) | birth weight >20th centile, no abnormal umbilical artery Doppler measurements, BP <140/90, no history of gestational diabetes, no histologic evidence of chorio-amnionitis | no | - | - | + |
| (19) | Hogg 2013a  Canada | cross-sectional | 37 (EOPE 19, LOPE 18) | 111 | 34.16±6.01 EOPE, 33.49±5.47 LOPE  vs.  33.10±4.74 | placenta | at the time of delivery | normotensive women with no placental pathology | Gestational age and gender matched subset 19 EOPE vs 19 controls | not reported | not reported | + |
| (20) | Hogg 2013b  Canada | cross-sectional | 37 (EOPE 19, LOPE 18) | 111 | 34.16±6.01 EOPE, 33.49±5.47 LOPE  vs.  33.10±4.74 | placenta | at the time of delivery | normotensive women with no placental pathology | no | not reported | not reported | + |
| (21) | Kim 2013  South Korea | cross-sectional | 22 | 161 | 34.3±3.9  vs.  32.8±3.7 | maternal peripheral blood (plasma)  placenta | not reported  at the time of delivery | women who delivered healthy normal neonates at term (>37 weeks of gestation) without medical or obstetric complications | no | - | not reported | + |
| (22) | Ruebner 2013  Germany | cross-sectional | 3 | 3 | Not reported | placenta | at the time of delivery | normotensive pregnant women | no | - | Not reported | + |
| (23) | Sundrani 2013  India | cross-sectional | 93 (term 48, preterm 45) | 46 | 23.1±2.9 term,  23.9±4.2 preterm  vs.  22.9±3.2 | placenta | at the time of delivery | normotensive singleton pregnancies | no | - | + | + |
| (24) | White 2013  USA | case-control | 14 | 14 | 23.3±4.3  vs.  24.9±2.9 | maternal peripheral blood | at the time of delivery (within 24h of delivery) | normotensive pregnant women | Age (±5 years), BMI (±5kg/m^2^), tobacco status (all non-smokers), and ethnicity | + | + | + |
| (25) | Xiang 2013a  China | cross-sectional | 16 | 16 | 30.95±6.48  vs.  28.5±3.73 | placenta | at the time of delivery | pregnancies undergoing Cesarean section withou suffering from other diseases | no | Not reported | Not reported | Not reported |
| (26) | Xiang 2013b  China | cross-sectional | 4  7  3 | 22  8  6 | 30.61±5.73  vs.  28.63±4.02 | placenta  umbilical cord blood  maternal peripheral blood | at the time of delivery  at the time of delivery  not reported | healthy women undergoing Cesarean section | no | Not reported | Not reported | Not reported |
| (27) | Yan 2013  China | cross-sectional | 30 | 30 | 28.5±3.8  vs.  27.9±3.0 | placenta | at the time of delivery | normotensive pregnant women who had operative delivery due to social factors or pelvic abnormalities | no | Not reported | + | Not reported |
| (28) | Anderson  2014^*^  USA | cohort | 6 | 6 | 22.78 (1.44)  vs.  27.5 (3.65) | maternal peripheral blood (WBC)  placenta | 1st trimester  at the time of delivery | pregnant normotensive nulliparous women >18 years who did not develop PE | Maternal age (range 0–9 years difference)  and weight at first prenatal visit (range 1–20 lb difference) | all nulliparous | not reported | + |
| (29) | Anton 2014  USA | case-control | 31 (term 19, preterm 12) | 14 | 27.9±7.7 PE, 28.0±8.1 term, 27.7±7.6 preterm  vs.  27.0±7.2 | placenta | at the time of delivery | women without hypertension-related complications that presented for delivery at term (≥37 gestational weeks) | no | not reported | not reported | unclear |
| (30) | Chu 2014  USA | cross-sectional | 24 | 24 | 27.9±7.2  vs.  29.3±5.4 | placenta | at the time of delivery | Women with uncomplicated (‘‘normal’’) pregnancy were normotensive and without proteinuria throughout gestation, and delivered healthy babies in the absence of infection | no | - | + | + |
| (31) | Liu 2014  China | cross-sectional | 21 | 16 | 30.6±1.0  vs.  31.6±1.4 | placenta | at the time of delivery | pregnant women with singleton gestation who underwent elective Cesarean section indicated by breech presentation or previous Cesarean section | Yes (no details) | not reported | + | + |
| (32) | Liu 2014  China | cross-sectional | 27 | 30 | 30.1±2.7  vs.  29.7±1.8 | placenta | at the time of delivery | normotensive term pregnancies | no | not reported | not reported | not reported |
| (33) | Liu 2014  China | case control | 12 | 12 | 28.00±4.88  vs.  26.50±3.03 | placenta | at the time of delivery | singleton uncomplicated pregnancies | Yes (no details) | - | + | + |
| (34) | Lu 2014  China | cross-sectional | 15 | 22 | 21-39 | placenta | 1st trimester - 6-9 weeks of gestation, during the procedure of induced abortion3rd trimester - at the time of delivery | 1st trimester normal pregnancy and normal full-term pregnancy | no | not reported | not reported | + |
| (35) | Ma 2014  USA | cross-sectional | 7 | 22 | Not reported | placenta | 1st trimester - 7-12 weeks of gestation, from legally induced abortions3rd trimester - at the time of delivery | 1st trimester normal pregnancy and normal full-term pregnancy | no | - | - | unclear |
| (36) | Nomura  2014^*^  USA | cohort | 5 | 45 | 24.4 (5.7) 15-41 | umbilical cord blood  placenta | at the time of delivery  at the time of delivery (within 6h) | pregnant women from low-income ethnic minority who did not develop PE | no | Not reported | Not reported | Not reported |
| (37) | Than 2014  USA | cross-sectional | 19 | 19 | 23 (21.0-27.0)  vs.  22 (20-28.5) | placenta umbilical cord  blood cells | at the time of delivery  at the time of delivery | preterm controls | Gestational age | Not reported | Not reported | + |
| (38) | Blair 2014  Canada | cross-sectional | 10 (5 PE of CMP16) | 10 | 35.6 (27-42)  vs.  33.7 (25-41) | placenta | at the time of delivery | chromosomally normal 3rd trimester pregnancies | Gestational age | Not reported | Not reported | Not reported |
| (39) | Rahat 2014  India | cross-sectional | 30 | 90 | 26±3.38  vs.  27.90±3.44 1^st^ trimester, 27.65±3.91 2^nd^ trimester, 28±3.5 3^rd^ trimester | placenta  maternal peripheral blood (WBC) | at the time of delivery  just before the procedure and 24 hours after for 3^rd^ trimester controls | 1^st^, 2^nd^ and 3^rd^ trimester healthy singleton pregnancies | no | Not reported | Not reported | Not reported |
| (40) | Ching 2014  Hawaii | case-control | 30 | 17 | 29.8±5.99  vs.  29.1±4.76 | placenta | at the time of delivery | full term pregnancies without preeclampsia | no | - | not reported | + |
| (41) | Anderson  2015^*^  USA | cohort | 3 | 3 | 25.3±0.72 (gestational hypertension group n=11)  vs.  24.2±0.62 | placenta | at the time of delivery | pregnant normotensive nulliparous women >18 years who did not develop PE | Yes (no details) | all nulliparous | not reported | + |
| (42) | Barrett 2015  Australia | case-control | 16 | 20 | 31.0 (1.6)  vs.  32.6 (1.0) | placenta | at the time of delivery | pregnant women in the 3rd trimester without PE | Maternal pregnancy BMI and gestational age at delivery | not reported | not reported | + |
| (43) | Ching 2015  Hawaii | case-control | 12 | 8 | 30.5±6.15  vs.  29.6±5.15 | umbilical cord blood | at the time of delivery | full term pregnancies without preeclampsia | no | - | not reported | + |
| (44) | Doridot 2015  France | cross-sectional | 5 | 5 | Not reported | placenta | at the time of delivery | women who underwent Cesarean surgery without suffering any disease during pregnancy | no | not reported | not reported | not reported |
| (45) | Ge 2015  China | cross-sectional | 127 | 132 | 29.1±4.3  vs.  28.5±3.2 | maternal peripheral blood (plasma)  placenta | on admission  at the time of delivery | normotensive women without PE | no | not reported | not reported | - |
| (46) | Hu 2015  China | cross-sectional | 10 | 10 | 29.1±2.9  vs.  28.5±4.7 | placenta | at the time of delivery | pregnant women without PE | no | not reported | not reported | not reported |
| (47) | Kim 2015  South Korea | nested case-control | 8 | 8 | 34.0 (31.6-36.0) EOPE, 34.1 (33.0-36.1) LOPE  vs.  33.0 (31.0-36.0) | placenta  maternal peripheral blood | at the time of delivery  at 6–41 gestational weeks | pregnant women without PE | no | - | - | + |
| (48) | Martin 2015  USA | cross-sectional | 19 | 17 | 28.4 (19-37)  vs.  28.2 (19-38) | placenta | at the time of delivery | normotensive pregnant women | no | - | - | + |
| (49) | Qi 2015  China | case-control | 45 | 45 | 30.5±2.84 mild, 29.3±4.7 severe  vs.  27.1±4.5 1^st^ trimester, 26.8±4 2^nd^ trimester, 26.8±4.3 3^rd^trimesterAll of | maternal peripheral blood (plasma) | on admission or at the time of diagnosis | normotensive pregnant women | no | Not reported | Not reported | + |
| (50) | Shan 2015  China | cross-sectional | 22 | 20 | 35.82±1.40  vs.  38.65±1.04 | placenta | 1st trimester - 6-8 weeks of gestation, after legal abortion for nonmedical reason normal  term and PE - at the time of delivery | healthy women undergoing abortion and normal term pregnancies | no | Not reported | Not reported | + |
| (51) | Shimanuki  2015  Japan | cross-sectional | 37 | 12 | 32 (27-45)  vs.  35 (19-47) | placenta | at the time of delivery | term normotensive pregnant women | Gestational period | Not reported | Not reported | + |
| (52) | Tang 2015  China | case-control | 19 | 20 | 30.53±4.67  vs.  30.70±5.10 | maternal peripheral blood (serum)  placenta | not reported  at the time of delivery | healthy women undergoing Cesarean section | no | Not reported | Not reported | + |
| (53) | Zhu 2015  China | cross-sectional | 20 | 20 | 27.75±4.27  vs.  26.25±1.26 | placenta | at the time of delivery | normal pregnant women | parity and gestation | - | Not reported | + |
| (54) | Jin 2016  China | cross-sectional | 6 | 6 | 29.83±3.82  vs.  30.17±2.32 | placenta | at the time of delivery | normotensive pregnant women with no history of chronic blood pressure elevation and an absence proteinuria | Yes (no details) | + | not reported | Not reported |
| (55) | Kim 2016  South Korea | cross-sectional | 6 | 6 | 32.3±5.4  vs.  31.6±2.4 | maternal peripheral blood (plasma)  placenta | soon after diagnosis of PE  at the time of delivery | normotensive women who provided clinical information (such as previous cesarean section, preterm labor, premature rupture of membranes) | Gestational age | not reported | not reported | + |
| (56) | Lin 2016  China | cross-sectional | 39 | 25 | 29.36±5.69 EOPE, 28.00±6.55 LOPE  vs.  28.32±4.55 | placenta | at the time of delivery | healthy pregnant women who delivered at full term | no | - | not reported | not reported |
| (57) | Rahat 2016a  India | cross-sectional | 30 | 90 | 26±3.3  vs.  27.9±3.4 1^st^ trimester, 27.6±3.9 2^nd^ trimester, 28±3.5 3^rd^ trimester | maternal peripheral blood (plasma and WBC)  placenta | before any obstetric procedure  1st and 2nd trimester - after elective termination of pregnancy, 3rd trimester - at the time of delivery | normal singleton pregnancy in the 1st, 2nd and 3rd trimester | no | Not reported | Not reported | Not reported |
| (58) | Rahat 2016b  India | cross-sectional | 30 | 90 | 26±3.3  vs.  27.9±3.4 1^st^ trimester, 27.6±3.9 2^nd^ trimester, 28±3.5 3^rd^ trimester | maternal peripheral blood (plasma and WBC)  placenta | before any obstetric procedure and 24h after delivery (only for normal 3rd trimester deliveries)  1st and 2nd trimester - after elective termination of pregnancy, 3rd trimester - at the time of delivery | normal singleton pregnancy in the 1st, 2nd and 3rd trimester | Gestational age | - | Not reported | Not reported |
| (59) | Rahat 2016c  India | cross-sectional | 30 | 90 | 26±3.38  vs.  27.90±3.44 1^st^ trimester, 27.65±3.91 2^nd^ trimester, 28±3.5 3^rd^ trimester | maternal peripheral blood (plasma and WBC)  placenta | before any obstetric procedure and 24h after delivery (only for normal 3rd trimester deliveries)  1st and 2nd trimester - after elective termination of pregnancy, 3rd trimester - at the time of delivery | normal singleton pregnancy in the 1st, 2nd and 3rd trimester | Gestational age | - | Not reported | Not reported |
| (60) | Wilson 2016  Canada | cross-sectional | 37 | 13 | Not reported | placenta | at the time of delivery | elective termination preterm birth due to various etiology with minimal placental involvement healthy term deliveries | no | Not reported | Not reported | unclear |
| (61) | Suzuki 2016  USA | cross-sectional | 36 | 15 | 26.6±6.1  vs.  27.3±3.3 | placenta | at the time of delivery | women without PE | no | - | - | unclear |
| (62) | White 2016  USA | case-control | 14 | 14 | 23.3±4.3  vs.  24.9±2.9 | maternal peripheral blood | at the time of delivery (within 24h of delivery) | normotensive pregnant women | Age (±5 years) and BMI (±5kg/m^2^) | + | + | + |
| (63) | Ye 2016  China | cross-sectional | 50 | 50 | 29.3 vs. 27.7 | maternal peripheral blood  placenta | one day before delivery  at the time of delivery | healthy pregnant women | Age and BMI | Not reported | Not reported | Not reported |
| (64) | Yeung 2016  Australia | cross-sectional | 8 | 16 | 28±2  vs.  32±1 | placenta | at the time of delivery | women without hypertension-related complications that presented for delivery at term (≥37 gestational weeks) | Gestational age | + | + | + |
| (65) | Herzog 2017  Netherlands | nested case-control | 29 (EOPE 13, LOPE 16) | 36 | 30 (4.7) EOPE, 33.3 (4.5) LOPE  vs.  31.8 (5.1) | umbilical cord WBC  placenta | at the time of delivery (with the placenta still in situ)  at the time of delivery | uncomplicated pregnancies | no | - | - | + |
| (66) | Jia 2017  China | cross-sectional | 12 | 14 | 37±0.39  vs.  38±0.24 | placenta | at the time of delivery | full term pregnant women without PE | no | not reported | not reported | not reported |
| (67) | Kim 2016  South Korea | nested case-control | 6 | 6 | 34.1 (32.7-36.0)  vs.  33.0 (31.0-35.3) | maternal peripheral blood  placenta | 6-14 and 15-23 weeks of gestation  During prenatal diagnostic procedures - 1st trimester and at the time of delivery - 3rd trimester | singleton pregnancies without PE | no | - | + | + |
| (68) | Liu 2017  China | case control | 16 | 16 | 27.14±3.77  vs.  25.83±4.18 | placenta | at the time of delivery | singleton uncomplicated pregnancies | Yes (no details) | - | + | + |
| (69) | Liu 2017  China | cross-sectional | 16 | 20 | 29.50±4.02  vs.  29.0±3.09 | placenta | at the time of delivery | term normotensive pregnant women | Yes (no details) | - | + | + |
| (70) | Majchrzak-Celinska 2017  Poland | case-control | 11 | 25 | 27.9 (7.5)  vs.  31.2 (4.8) | placenta | at the time of delivery (within 2 hours of delivery) | normotensive pregnant women without proteinuria who fulfilled the inclusion criteria | no | - | Not reported | + |
| (71) | Sari 2017  Turkey | cross-sectional | 260 | 260 | 29±7.03 (16-48)  vs.  27.9±5.63 (16-42) | maternal peripheral blood (lymphocytes) | not reported | healthy 20 or more weeks pregnant women without chronic hypertension and diabetes | no | - | - | + |
| (72) | Van den Berg 2017  Netherlands | nested case-control | placenta 22umbilical cord blood 22 | placenta 25umbilical cord blood 25 | 30.0±4.7 EOPE, 33.3±4.5 LOPE  vs.  31.8±5.1 | placenta  umbilical cord blood | at the time of delivery  at the time of delivery | uncomplicated and complicated controls (FGR and spontaneous PTB) | no | - | - | + |
| (73) | Xiao 2017  China | cross-sectional | 19 | 10 | 31.9±2.6  vs.  29.6±4.6 | placenta | at the time of delivery | normal pregnant women choosing Cesarean delivery according to their personal request or had a history | no | Not reported | Not reported | + |
| (74) | Zhao 2017  China | cross-sectional | 19 | 17 | 28.4±5.7  vs.  28.2±5.9 | placenta | at the time of delivery | normotensive pregnant women | no | - | - | Not reported |
| (75) | Rahat 2017  India | cross-sectional | 30 | 90 | 26±3.3  vs.  27.9±3.4 1^st^ trimester, 27.6±3.9 2^nd^ trimester, 28±3.5 3^rd^ trimester | Placenta  maternal peripheral blood (WBC) | at the time of delivery  48 hours after delivery | 1^st^, 2^nd^ and 3^rd^ trimester healthy singleton pregnancies | Gestational age | Not reported | Not reported | Not reported |
| (76) | Saraswathy 2017  India | cross-sectional | 103 | 616 | Not reported | maternal peripheral blood (plasma) | not reported | normal healthy controls were women without any systemic or obstetric complications during the entire period of gestation | no | Not reported | Not reported | + |
| (77) | Ma 2018  China | cross-sectional | 10 | 10 | 29.37±0.74  vs.  28.33±0.62 | placenta | at the time of delivery | healthy pregnant women in 3rd trimester | no | not reported | not reported | unclear |
| (78) | Mohammadpour -Gharehbagh 2018  Iran | case-control | 54 | 55 | 28.4±6.9  vs.  29.4±6.3 | placenta | at the time of delivery | normotensive pregnant women | no | - | Not reported | + |
| (79) | Rezaei 2018  Iran | case-control | 104 | 119 | 29.3±7.6  vs.  27.5±6.3 | placenta | at the time of delivery | normotensive pregnant women | no | - | Not reported | + |
| (80) | Wilson 2018  Canada | cross-sectional | 40 (EOPE 22, LOPE 18) | 43 (term control 24, preterm control 19) | 19.7-42.9 (33.3) EOPE, 23.1-41.3 (34.0) LOPE  vs.  22.2-41.1 (32.5) term control, 30.0-40.2 (34.9) | placenta | at the time of delivery | normotensive pregnant women | Gestational age | Not reported | Not reported | + |
| (81) | Alahari 2018  Canada | case-control | 6-8 | 6-8 | Not reported | placenta | at the time of delivery | Preterm and term normotensive age-matched deliveries | Age | Not reported | Not reported | Not reported |
| (82) | Li 2018  China | cross-sectional | 10 | 11 | 30.97 (22-40) vs.  31.36 (28-32) | placenta | at the time of delivery | normal pregnancy controls | no | Not reported | Not reported | Not reported |
| (83) | Zhu 2018  China | cross-sectional | 10 | 10 | 27.83±3.27  vs.  26.76±2.45 | placenta | at the time of delivery | normal pregnant women | no | Not reported | Not reported | + |
| (84) | Zhang 2018  China | cross-sectional | 25 | 50 | 29.92±6.24  vs.  31.06±5.25 | placenta | at the time of delivery | normotensive term and preterm pregnancies | no | Not reported | + | + |
| (85) | Fan 2019  China | case-control | 42 | 45 | 28.3±4.2  vs.  28.1±4.6 | placental vessels | at the time of delivery | blood pressure < 120/90 mm Hg with no significant complications | no | Not reported | Not reported | Not reported |
| (86) | Gao 2019  China | cross-sectional | 40 | 42 | 28.20±4.10  vs.  28.40±4.50 | umbilical vein | at the time of delivery | healthy pregnant participants defined as blood pressure < 120/90 mmHg and no clinically significant complications | no | Not reported | Not reported | + |
| (87) | Halvatsiotis 2019  Greece | cross-cestional | 6 | 6 | 33 (27-39)  vs.  31.5 (25-38) | maternal peripheral blood | Not reported | pregnant women without any history of autoimmunity, malignancy, or family history for GDM and PE (volunteers) | no | Not reported | Not reported | Not reported |
| (88) | Mishra 2019a  India | case-control | 40 | 39 + 26 (gestational matched + full term) | Not reported | maternal peripheral blood (WBC)  placenta | not reported | gestation matched and full term pregnant women | Gestation and age | Not reported | Not reported | Not reported |
| (89) | Wang 2019  China | cross-sectional | 22 | 20 | 31.23±5.26  vs.  30.09±3.97 | placenta | at the time of delivery | pregnancies without PE, gestational hypertension, fetal growth restriction or preterm birth (*<*37 weeks) | no | Not reported | Not reported | Not reported |
| (90) | Mohammadpour-Gharehbagh 2019  Iran | cross-sectional | 92 | 106 | 28.5±6.8  vs.  29.7±6.4 | placenta | At the time of delivery | normotensive pregnant women | no | Not reported | Not reported | Not reported |

^*^Population for four cohort studies: Anderson 2015 (41) - pregnant normotensive nulliparous women >18 years in the first trimester of pregnancy from a local community; Anderson 2014 (28) - pregnant normotensive nulliparous women >18 years in the first trimester of pregnancy from a local community; Bourque 2010 (5) - past history of preeclampsia (severe, early onset and/or associated with perinatal loss), pre-existing hypertension, unexplained low first trimester PAPP-A (<0.60 multiples of the median [MoM]), unexplained elevated second trimester alpha-fetoprotein (AFP; >2.5 MoM) or human chorionic gonadotrophin (HCG;> 3.0 MoM); Nomura 2014 (36)-pregnant women from low-income ethnic minority

Abbreviations: WBC – white blood cells; CMP16 - confined placental mosaicism for chr. 16

Age is expressed as mean±sd, mean (se), median (range)

Table S2. Definitions of preeclampsia

| **Definition** | **Author/Guideline** | **Ref.** |
| --- | --- | --- |
| PE was defines as 1) Systolic blood pressure≥140 mmHg or diastolic blood pressure≥90 mmHg with proteinuria≥300 mg/24 h after 20 weeks of gestation; 2) Systolic blood pressure≥140 mmHg or diastolic blood pressure≥90 mmHg without proteinuria, but with pregnancy complications such as earlyonset thrombocytopenia, impaired liver function, impaired kidney function, pulmonary edema and damaged vision or brain | American College of Obstetricians and Gynecologists **(ACOG) 2013** | (53,71,73,77,79,81,84,85,89,90) |
| PE was defined as the increase in blood pressure to ≥140/90 mmHg at 20 weeks after gestation, accompanied by urinary protein excretion (300 mg protein in a 24-h urine specimen). Severe PE was defined as either severe hypertension (systolic blood pressure of ≥160 mmHg and/or diastolic blood pressure of ≥110 mmHg on ≥2 occasions 6 h apart) plus mild proteinuria or mild hypertension plus severe proteinuria (>2 g/24 h). | American College of Obstetricians and Gynecologists **(ACOG) 2002**  National High Blood Pressure Education Program Working Group on High Blood Pressure in Pregnancy | (13,18,29,34,37,49,66,67)  (13,30,31) |
| Not reported | the American Congress of Obstetricians and Gynecologists (no reference, no year) | (35,48,50,52) |
| Preeclampsia was defined as: (1) at least two of the following: hypertension (sBP ≥140 mmHg and/or dBP ≥90 mmHg, twice, >4 h apart) after 20 weeks, and proteinuria defined as ≥0.3 g/d or ≥2+ dipstick proteinuria after 20 weeks, (2) non-hypertensive and non-proteinuric HELLP syndrome, using Sibai’s criteria, or (3) an isolated eclamptic seizure without preceding hypertension or  proteinuria, using the British Eclampsia Survey Team (BEST) criteria to define eclampsia | Canadian guideline 2008  Audibert 1996  Douglas KA 1994 | (5,7)  (17,19,20,80) |
| Severe PE was diagnosed as patients who had no history of preexisting or chronic hypertension, but showed systolic blood pressure of >160mmHg or diastolic blood pressure of >110mmHg on at least two occasions, accompanied by significant proteinuria (<2 g per 24 h or 3+ by dipstick in two random samples collected at 4 h interval) after 20 weeks of gestation | The International Society for the Study of Hypertension in Pregnancy - **ISSHP** | (10,65,72) |
| Preeclampsia was diagnosed in gravid females by new onset of hypertension (systolic blood pressure of 140 mm Hg and/or diastolic blood pressure 90 mm Hg) and proteinuria (300 mg or more of protein in the urine per 24-hour collection) that occurred in women who were otherwise normal | Cunningham FG 2005 | (14–16) |
| Preeclampsia was defined as gestational hypertension with proteinuria. Gestational hypertension was defined as persistent, new onset hypertension (systolic ≥140 mm Hg and/or diastolic ≥90 mmHg) appearing after 20 weeks’ gestation. Proteinuria was defined as ≥300 mg of protein in a 24 hour urine collection, a dipstick of 2+, a catheterized sample of 1+, or protein:creatinine ≥0.3. To ensure that preeclamptic women had the specific disease of interest, our research definition also required gestational hyperuricemia (≥1 standard deviation above reference values for the gestational age (e.g. term, .5.5 mg/dL)) | The National High Blood Pressure Education Program Working Group Report on High Blood Pressure in Pregnancy 2000  Chesley LC 1980 | (30,51,68) |
| PE group was defined as severe PE according toone or more of the following criteria: maternal blood pressure≥160/110 mm Hg on 2 separate readings; proteinuria >2+ by dipstickor >2 g/24 hours; visual disturbances; pulmonary edema;epigastric or right upper quadrant pain; or fetal growth restriction. | Steegers 2010 Lancet | (66,86,87) |
| Not reported | Society of Obstetric Medicine, Australia New Zealand guidelines research definition (**ANZJOG**) | (42,64) |
| Pre‐eclamptic pregnancies were defined by the onset of hypertension during pregnancy (blood pressure ≥ 140/90 mm Hg, with no prior hypertension history) and consistent proteinuria (≥300 mg/d) | Young BC 2010  Steegers E, 2010 Lancet | (86,87) |
| PE was defined as systolic pressure >140 mm Hg, diastolic pressure >90 mmHg and proteinuria ≥0.3 g/24 h | Not reported | (2,9,44) |
| Preeclampsia was defined as the presence of new onset hypertension (>140/90mmHg), as documented by at least two readings 6 h apart, accompanied by proteinuria, as defined by a 24 h urine protein excretion >300 mg, or the equivalent based on either the protein/creatinine ratio or urine dipstick | Not reported | (24,62) |
| PE patients were as follows: systolic pressure >140 mmHg, diastolic pressure >90 mmHg, and proteinuria >0.3 g in a 24 hours collection | Not reported | (12,25) |
| Pregnant women diagnosed with clinical symptoms of systolic pressure of 140 mm Hg and diastolicpressure of 90 mm Hg, proteinuria >300 mg in 24 h | Not reported | (58,59) |
| The diagnosis of IUGR, PE and HELLP was based on general accepted criteria  previously described in Langbein et al. (2008) | Langbein et al. 2008 | (22) |
| PE was defined as documented diagnosis or evidence of new-onset hypertension (systolic blood pressure ≥140 mmHg or diastolic BP ≥90 mmHg) combined with proteinuria (≥+1 single sample or > 300 mg/24-hr urine sample) during the second half of pregnancy or during the postpartum hospitalization | Roberts et al. 2003 | (28) |
| Severe preeclampsia was defined as severe hypertension that developed after 20 weeks (systolic or diastolic blood pressure ≥160 or ≥110mmHg, respectively, measured at two different time points, 4h to 1 week apart) coupled with proteinuria (≥300mg in a 24h urine collection, or two random urine specimens obtained 4h to 1 week apart containing ≥1+ by dipstick or one dipstick of ≥2+ protein) | Sibai 2005 | (37) |
| Not reported | Yang 2016 | (39) |
| Not reported | Le J (2008). Obstetrics and Gynecology. 7th edn. (Le J, eds.). People’s Medical Publishing House, Beijing, 92-99. | (45) |
| Not reported | Roberts 1993 | (46) |
| The study definition for Preeclampsia was presence of hypertension (blood pressure ≥140/90 mmHg) associated with proteinuria (≥0.3g/24hrs) | Chaiworapongsa T et al. 2014 | (82) |
| PE was defined by the International Society for the Study of Hypertension in Pregnancy were taken up for the investigation: those with systolic blood pressure P140 mm of Hg and diastolic blood pressure P90 mmHg (confirmed by 2 readings 4–6 h apart) and proteinuria P300 mg/24 h urine from 20 weeks of gestation | S. Grill, C. Rusterholz, R. Zanetti-Dallenbach, et al., Potential markers of preeclampsia  – a review, Reprod. Biol. Endocrinol. 7 (2009) 70–84. | (83) |
| severe preeclampsia (diastolic blood pressure >110 mmHg and 3+ proteinuria) | Not reported | (1) |
| Mild pre-eclampsia was defined as a systolic blood pressure >140 mmHg or diastolic blood pressure >90 mmHg on at least two occasions after 20 weeks of gestation with the presence of proteinuria defined as >0.3 g/day. Severe pre-eclampsia was defined as a systolic pressure >160 mmHg or diastolic pressure 110 mmHg on at least two occasions after 20 weeks of gestation with the presence of severe proteinuria defined as >5 g/day | Not reported | (3) |
| PE was defined as systolic pressure ≥140 mmHg, diastolic pressure ≥90 mmHg, and proteinuria ≥ 0.3 g per 24 h, occurring after 20 weeks of gestation in a woman with previously normal blood pressure | Not reported | (6) |
| Pre-eclampsia was defined by systolic and diastolic blood pressures >140 and 90mm Hg, respectively, with the presence of proteinuria (>1+ or 300 mg/24 h) on a dipstick test | Not reported | (11) |
| PE was defined as hypertension (systolic blood pressure ≥140 mmHg and/or diastolic blood pressure ≥90 mmHg) and proteinuria ( ≥300 mg in a 24 h urine collection and/or ≥1+ on dipstick testing) after 20 weeks of gestation | Not reported | (21) |
| PE was defined by systolic and diastolic blood pressures greater than 140 and 90 mm Hg, respectively, with presence of proteinuria (>1+ or 300 mg/24 hrs) on a dipstick test and was confirmed by repeated recording of the blood pressure with an interval of 6 hrs | Not reported | (23) |
| Pre-eclampsia was defined as a blood pressure of ≥ 140/90 mmHg taken twice, 6 hours apart, with proteinuria of ≥ 2+ or ≥ 300 mg in a 24- hour collection. | Not reported | (32) |
| PE was defined as new onset hypertension developed in the second half of pregnancy, defined as systolic blood pressure >140 mm Hg or diastolic blood pressure >90 mm Hg on 2 separate occasions, at least 6 h apart, coupled with proteinuria (>300 mg/24 h or ≥+1 protein obtained on an independent urine sample measurement) | Not reported | (41) |
| PE was defined as hypertension (systolic blood pressure ≥140mmHgand/or  diastolic blood pressure ≥90 mmHg, twice, 4h apart) and proteinuria (≥0.3 g/day urine collectionand/or ≥1+ on dipstick testing) after 20 weeks of gestation | Not reported | (47) |
| PE was defined as gestational hypertension (systolic blood pressure ≥140 mmHg  or diastolic blood pressure ≥90 mmHg measured twice, at least 4 h apart) arising after 20 weeks of gestation and proteinuria (≥300 mg/day or ≥2+ dipstick) or other adverse conditions. | Not reported | (55) |
| 140/90 mmHg systolic/diastolic pressure; proteinuria >300 mg in 24 h, or 1+ protein on dipstick | Not reported | (57) |
| Diagnosis of PE was defined as follows: sustained systolic blood pressure of ≥140 mmHg or sustaineddiastolic blood pressure of ≥90 mmHg on 2 separate readings; proteinuria measurement of 1+ or moreon a dipstick; or a 24-h urine protein collection with 300 mg in specimen or observed other PE clinicalcharacteristics | Not reported | (61) |
| The preeclampsia was determined as severe if BP ≥160/110 mmHg and proteinuria ≥2g/24 h, orsymptoms of organ damage appeared such as headache and thrombocytopenia; otherwise it was determined to be mild. The onset of the preeclampsia was determined as early if it occurred at less than 34 weeks of gestational age, and otherwise as late. | Not reported | (63) |
| PE was defined as (i) women had a blood pressure ≥140 mmHg systolic and/or ≥90 mmHg diastolic after 20 wk gestation on two occasions at least 4 h apart; (ii) proteinuria 1+ or ≥300 mg in 24 h orrenal insufficiency (serum creatinine >0.09 mmol/l) | Not reported | (69) |
| Patients were diagnosed with PE after having an episode of hypertension  (with consistent blood pressure of 140/90 mm Hg) and newly onset proteinuria >0.3 g/24 h. | Not reported | (70) |
| PE was defined as clinical symptoms of systolic pressure 140 mmHg and diastolic pressure 90 mmHg, proteinuria >300 mg in 24 h | Not reported | (75) |
| Preeclampsia detection was based increased systolic blood pressure (SBP) ≥140mmHg and diastolic blood pressure (DBP) ≥90mm Hg on 2 or more measurements at least 6 h apart. The other including criteria was dipstick urine proteinuria (≥0.3 g/24h or ≥+1) after 20 weeks of gestation | Not reported | (78) |
| PE was defines as systolic blood pressure (SBP) ≥ 140 or diastolic blood pressure (DBP) ≥ 90 on two occasions at least 4 h apart, and proteinuria on dipstick ≥1 or ≥ 30 mg/24 h after 20 gestational weeks | Not reported | (88) |

References number (4,8,12,27,33,36,38,40,43,54,56,60,74,76) didn`t report definition for preeclampsia

Table S3. Criteria for less severe and more severe forms of preeclampsia

| **Study** | **Less severe form** | **Criteria** | **More severe form** | **Criteria** |
| --- | --- | --- | --- | --- |
| Anderson 2014 | LOPE | >34 weeks of gestation | EOPE | ≤34 weeks of gestation |
| Anton 2014 | LOPE | >37 weeks of gestation | EOPE | ≤37 weeks of gestation |
| Blair 2013 | NA | NA | EOPE | Resistant hypertension, or new or worsening proteinuria, or One/more adverse condition(s): maternal symptoms (persistent or new/unusual headache, visual disturbances, persistent abdominal or right upper quadrant pain, severe nausea or vomiting, chest pain or dyspnea), maternal signs of end-organ dysfunction (eclampsia, severe hypertension, pulmonary edema, or suspected placental abruption), abnormal maternal laboratory testing (elevated serum creatinine [according to local laboratory criteria]; elevated AST, ALT or LDH [according to local laboratory criteria] with symptoms; platelet count < 20 g/L), or fetal morbidity (oligohydramnios, intrauterine growth restriction, absent or reversed end-diastolic flow in the umbilical artery by Doppler velocimetry, or intrauterine fetal death) |
| Chelbi 2011 | NA | NA | EOPE | systolic pressure >140 mm Hg, diastolic pressure >90 mm  Hg and proteinuria ≥0.3 g/24 h before 34 weeks of gestation |
| Ching 2015 | NA | NA | EOPE | <34 weeks of gestation |
| Ching 2014 | NA | NA | EOPE | <34 weeks of gestation |
| Czikk 2013 | NA | NA | sPE | Defined as: 1) Systolic BP >140 systolic or >90 diastolic AND proteinuria of >1+ on dipstick or >300 mg/24 h AND delivery under 34+6 weeks, with a birth weight >10th percentile OR 2) Systolic BP >160 mmHg or a diastolic BP >110 mmHg AND proteinuria of >1+ on dipstick or >300 mg/24 h AND delivery at less than 34+6 weeks, with a birth weight >10^th^ percentile. |
| Gao 2011 | LOPE | >34 weeks of gestation | EOPE | ≤34 weeks of gestation |
| Ge 2015 | LOPE | >34 weeks of gestation | sPE  EOPE | Severe preeclampsia: no history of preexisting or chronic hypertension, but showed systolic blood pressure of 4160mmHg or diastolic blood pressure of 4110mmHg on at least two occasions, accompanied by significant proteinuria (42 g per 24 h or 3+ by dipstick in two random samples collected at 44 h interval) after 20 weeks of gestation.  ≤34 weeks of gestation |
| Herzog 2017 | LOPE | >34 weeks of gestation | EOPE | ≤34 weeks of gestation |
| Hogg 2013a | LOPE | >34 weeks of gestation | EOPE | ≤34 weeks of gestation |
| Hogg 2013b | LOPE | >34 weeks of gestation | EOPE | ≤34 weeks of gestation |
| Jia 2012 | NA | NA | sPE | Either severe hypertension (systolic blood pressure of ≥160 mmHg and/or diastolic blood pressure of ≥110 mmHg on ≥2 occasions 6 h apart) plus mild proteinuria or mild hypertension plus severe proteinuria (>2 g/24 h). |
| Jia 2017 | NA | NA | sPE | Either severe hypertension (systolic blood pressure of ≥160 mmHg and/or diastolic blood pressure of ≥110 mmHg on ≥2 occasions 6 h apart) plus mild proteinuria or mild hypertension plus severe proteinuria (>2 g/24 h). |
| Kim 2015 | LOPE | >34 weeks of gestation | EOPE | ≤34 weeks of gestation |
| Lin 2016 | LOPE | >34 weeks of gestation | EOPE | ≤34 weeks of gestation |
| Liu 2014 | NA | NA | sPE | Severe PE was defined according to National High Blood Pressure Education Program Working Group, 2000 - AJOG |
| Lu 2014 | NA | NA | sPE | blood pressure of ≥160/110 mmHg on 2 occasions at least 6 h apart after 20 weeks of gestation and proteinuria (≥5 g/24 h or ≥3+ on 2 random urine samples collected at least 4 h apart) |
| Mousa 2012b | mPE | blood pressure was ≥140 mmHg systolic or ≥90 mmHg diastolic and there was 0.3 g of urinary protein per 24 hours after 20 weeks of gestation. | sPE | blood pressure ≥160 mmHg systolic or ≥110 mmHg diastolic, with 5 g of protein in the urine within 24 hours after 20 weeks of gestation |
| Muller 2004 | NA | NA | sPE | diastolic blood pressure ≥110 mmHg and 3+ proteinuria |
| Mohammadpour-Gharehbagh 2017 | NA | NA | sPE | higher blood pressure (SBP ≥ 160mmHg or DBP ≥ 110mmHg) or severe proteinuria (≥5 g protein in a 24 h urine collection) |
| Qi 2015 | mPE | blood pressure ≥140/90 mmHg at ≥20 weeks’  gestation, 1+ urine protein or ≥300 mg/24 h, and absence  of concomitant symptoms such as epigastric discomfort,  headache, and blurred vision | sPE | Presence of one of more of the following signs or symptoms in addition to fulfilling the diagnosis of mild preeclampsia: blood pressure ≥160/110 mmHg; platelet count ≤100 9 109/L; increased serum transaminases; central nervous system dysfunction including blurred vision, headache, dizziness, or even unconsciousness and coma; epigastric discomfort or persistent upper abdominal pain; oliguria: urine output <500 mL/ 24 h; lung edema; cerebrovascular accident; intravascular hemolysis: anemia, jaundice, or increase in lactate dehydrogenase; coagulation dysfunction; fetal growth restriction or oligohydramnios |
| Rezaei 2018 | NA  LOPE | NA  >34 weeks of gestation | sPE | higher blood pressure (SBP ≥ 160 mmHg or DBP ≥ 110 mmHg) or severe proteinuria (≥5 g protein in a 24 h urine collection)  ≤34 weeks of gestation |
| Robinson 2016 | LOPE | >34 weeks of gestation | EOPE | ≤34 weeks of gestation |
| Shimanuki 2015 | LOPE | >34 weeks of gestation | EOPE | ≤34 weeks of gestation |
| Tsui 2007 | mPE | Systolic blood pressure *>*140 mmHg or diastolic blood pressure *>*90 mmHg on at least two occasions after 20 weeks of gestation with the presence of proteinuria defined as *>*0*.*3 g/day. | sPE | systolic pressure *>*160 mmHg or diastolic pressure  110 mmHg on at least two occasions after 20 weeks of  gestation with the presence of severe proteinuria defined  as *>*5 g/day. |
| Wilson 2018 | LOPE | >34 weeks of gestation | EOPE | ≤34 weeks of gestation |
| Mohammadpour‑Gharehbagh 2018 | NA | NA | sPE | either higher blood pressure (SBP ≥ 160mmHg or DBP ≥ 110mmHg) or severe proteinuria (≥5 g protein in a 24 h urine collection) |
| Ye 2015 | mPE  LOPE | Otherwise  >34 weeks of gestation | sPE  EOPE | BP _160/110 mmHg and proteinuria _2g/24 h, or  symptoms of organ damage appeared such as headache and  thrombocytopenia  ≤34 weeks of gestation |
| Yuen 2010 | LOPE | >34 weeks of gestation | EOPE | ≤34 weeks of gestation |
| Zhu 2015 | NA | NA | sPE | ACOG criteria |
| Zhang 2019 | NA | NA | EOPE | ≤34 weeks of gestation |
| Wang 2019 |  |  | EOPE | EOPE was diagnosed by new-onset gestational hypertension of at least 140/90 mmHg on two occasions at least 4 h apart accompanied by proteinuria ≥300 mg/day from 24 h urine collection at ≥20 weeks and |
| Zhang 2018 | NA | NA | EOPE | before 34 weeks of gestation |
| Mohammadpour-Gharehbagh 2019 | NA | NA | sPE | SBP ≥ 160 mmHg or DBP ≥ 110 mmHg on two occasions at least 4 h apart while the patient is on bed rest |

Abbreviations: EOPE-early onset preeclampsia; LOPE-late onset preeclampsia; mPE - mild preeclampsia; sPE – severe preeclampsia

Ref (5,23,32,59,69,70,75,76) - didn`t report criteria for PE

Ref Alahari 2018 didn`t report criteria for EOPE and LOPE, Ref Li 2018 didn`t report criteria for sPE

Table S4. Exclusion criteria for studies examining DNA methylation between preeclamptic and normotensive women

| **ref** | **Study** | **Smoking** | **Chronic HTN** | **CVD** | **Diabetes** | **Renal Disease** | **Obesity** | **Previous PE** | **Other Exclusion Criteria** | **Other Inclusion Criteria** |
| --- | --- | --- | --- | --- | --- | --- | --- | --- | --- | --- |
| (5) | Bourque 2010 | Not reported | no | Not reported | Not reported | Not reported | Not reported | yes | Not reported | unexplained low first trimester PAPP-A (<0.60 multiples of the median [MoM]), unexplained elevated second trimester alpha-fetoprotein (AFP; >2.5 MoM) or human chorionic gonadotrophin (HCG; > 3.0 MoM) |
| (7) | Yuen 2010 | Not reported | yes | Not reported | Not reported | Not reported | Not reported | Not reported | Low birth weight | Not reported |
| (8) | Zhao 2010 | Not reported | Not reported | yes | Not reported | yes | Not reported | Not reported | Other hypertension-associated diseases | Not reported |
| (9) | Chelbi 2011 | yes | yes | Not reported | yes | yes | yes | Not reported | Multiple pregnancies | Not reported |
| (10) | Gao 2011 | Not reported | yes | Not reported | Not reported | Not reported | Not reported | Not reported | Women who developed renal disease, transient hypertension in pregnancy, gestational diabetes, spontaneous abortion, intrauterine fetal death, fetal chromosomal or congenital abnormalities, pregnancies conceived by fertility treatment | Not reported |
| (11) | Kulkarni 2011 | yes | Not reported | Not reported | Not reported | Not reported | Not reported | Not reported | Pregnancy complications such as multiple gestations, chronic hypertension, type I or type II diabetes, seizure disorder, renal or liver disease, alcohol or drug abuse | Singleton pregnancies |
| (12) | Zhao 2011 | Not reported | Not reported | yes | Not reported | yes | Not reported | Not reported | Other hypertension associated diseases | Not reported |
| (13) | Jia 2012 | Not reported | yes | yes | Not reported | Not reported | Not reported | Not reported | Chronic illness or long term use of medications, multiple pregnancies, premature rupture of membranes and fetal anomalies | Not reported |
| (14) | Mousa 2012a | yes | Not reported | Not reported | yes | Not reported | Not reported | Not reported | chorioamnionitis, maternal infections, active sexually transmitted diseases, lupus | Not reported |
| (15) | Mousa 2012b | yes | Not reported | Not reported | yes | Not reported | Not reported | Not reported | chorioamnionitis, maternal infections, active sexually transmitted diseases, lupus | Not reported |
| (19) | Hogg 2013a | Not reported | Not reported | Not reported | Not reported | Not reported | Not reported | Not reported | Gestational diabetes, isolated maternal hypertension, non-singleton pregnancies, still birth, fetal genetic anomaly | Women between the age of 18 and 42 years |
| (20) | Hogg 2013b | Not reported | Not reported | Not reported | Not reported | Not reported | Not reported | Not reported | Gestational diabetes, isolated pregnancy induced hypertension, intrauterine death and fetal genetic anomalies | Women between the age of 18 and 42 years |
| (21) | Kim 2013 | Not reported | yes | Not reported | yes | yes (chronic kidney disease) | Not reported | Not reported | Liver disease | Not reported |
| (22) | Ruebner 2013 | Not reported | Not reported | Not reported | yes | Not reported | Not reported | Not reported | Cancer | Not reported |
| (23) | Sundrani 2013 | Not reported | Not reported | Not reported | Not reported | Not reported | Not reported | Not reported | Pregnancy complications such as chronic hypertension, type I or II diabetes mellitus, seizure disorder, renal or liver disease | Singleton pregnancies |
| (24) | White 2013 | yes | Not reported | Not reported | Not reported | Not reported | Not reported | Not reported | Not reported | First pregnancy, European descent |
| (25) | Xiang 2013a | Not reported | Not reported | Not reported | Not reported | Not reported | Not reported | Not reported | Not reported | Han ethnicity |
| (27) | Yan 2013 | yes | Not reported | Not reported | Not reported | Not reported | Not reported | Not reported | Other pregnancy complications, multiple pregnancies, infectious diseases, drug addiction, congenital deformities of the fetus | 3rt trimester pregnant women |
| (28) | Anderson 2013 | Not reported | Not reported | Not reported | Not reported | Not reported | Not reported | Not reported | Not reported | Nulliparous women in the 1^st^ trimester pregnancy from a local community, aged >18 years in the first trimester (<14 completed weeks) |
| (30) | Chu 2014 | yes | Not reported | yes (vascular disease) | Not reported | yes | Not reported | Not reported | Not reported | Not reported |
| (31) | Liu 2014a | yes | Not reported | yes | Not reported | yes | Not reported | Not reported | Other hypertension-associated diseases, alcohol abuse | singleton gestations who underwent elective cesarean section indicated by PE, breech presentation, or previous cesarean section |
| (33) | Liu 2014c | yes | yes | Not reported | yes | yes | Not reported | Not reported | Intrapartum infection, chrioamnionitis, gestational diabetes mellitus, premature rupture of membranes, alcohol abuse, long-term use of medications, assisted reproduction | Not reported |
| (34) | Lu 2014 | Not reported | yes | Not reported | yes | yes (nephropathy) | Not reported | Not reported | Recent urinary tract infection | 21-39 years old |
| (35) | Ma 2014 | Not reported | Not reported | Not reported | Not reported | Not reported | Not reported | Not reported | Serious maternal complications and fetal abnormalities | Not reported |
| (36) | Nomura 2014 | Not reported | Not reported | Not reported | Not reported | Not reported | Not reported | Not reported | HIV infection, maternal psychosis, maternal age <15 years, life-threatening medical complications of the mother, congenital or chromosomal  abnormalities of the fetus | Not reported |
| (39) | Rahat 2014 | Not reported | Not reported | Not reported | Not reported | Not reported | Not reported | Not reported | Not reported | Singleton pregnancies |
| (40) | Ching 2014 | Not reported | yes | Not reported | yes | yes | Not reported | Not reported | Gestational diabetes mellitus, hyperthyroidism, systemic lupus erythematous, twin pregnancy | Not reported |
| (41) | Anderson 2014 | Not reported | Not reported | Not reported | Not reported | Not reported | Not reported | Not reported | Not reported | nulliparous women, aged  >18 years in the first trimester (<14 completed weeks) of pregnancy |
| (42) | Barret 2015 | Not reported | Not reported | Not reported | Not reported | Not reported | Not reported | Not reported | Not reported | Third trimester pregnancy women |
| (43) | Ching 2015 | Not reported | yes | Not reported | yes | yes | Not reported | Not reported | Gestational diabetes mellitus, , hyperthyroidism, systemic lupus erythematous | Singleton pregnancies |
| (45) | Ge 2015 | Not reported | yes (primary HTN) | yes | yes | yes (chronic nephritis) | Not reported | Not reported | Not reported | Han women |
| (46) | Hu 2015 | Not reported | Not reported | Not reported | Not reported | Not reported | Not reported | Not reported | Not reported | Chinese Han pregnant women who were not taking regular medication |
| (47) | Kim 2015 | Not reported | yes | Not reported | yes | yes (chronic kidney disease) | Not reported | yes | Liver disease | Singleton pregnancies |
| (48) | Martin 2015 | Not reported | Not reported | Not reported | x (pre-diabetes and diabetes) | Not reported | Not reported | Not reported | Gestational diabetes mellitus | Not reported |
| (49) | Qi 2015 | Not reported | yes | Not reported | yes | Not reported | Not reported | Not reported | Not reported | Han ethnicity |
| (50) | Shan 2015 | Not reported | yes | yes | yes | yes (chronic renal disease) | Not reported | Not reported | Collagen disorders, metabolic diseases | Not reported |
| (53) | Zhu 2015 | Not reported | yes | yes (cardiac insufficiency) | yes | yes | Not reported | Not reported | Multiple pregnancies, HIV infection, preterm | Han Chinese women who were delivered by elective caesarean section |
| (54) | Jin 2016 | Not reported | Not clear | yes | yes | yes | Not reported | Not reported | Cancer | Pregnant women over 18 and less than 45 years |
| (55) | Kim 2016 | Not reported | yes | Not reported | Not reported | yes | Not reported | Not reported | Multiple gestations, gestational hypertension without proteinuria, nephritic syndrome diagnosed postpartum or other major metabolic disorders | Not reported |
| (56) | Lin 2016 | Not reported | Not reported | Not reported | yes | yes (kidney disease) | Not reported | Not reported | Stillbirth and fetal malformation, immune disease, cancer, epilepsy, other obstetrical complications, multiple pregnancies (≥3 fetuses), alcohol and drug addiction | Not reported |
| (58) | Rahat 2016b | Not reported | Not reported | Not reported | Not reported | Not reported | Not reported | Not reported | Gestational diabetes mellitus, gestational trophoblast disease, IUGR, Rh- negative isoimmunised women, thalassemia | Not reported |
| (60) | Robinson 2016 | Not reported | Not reported | Not reported | Not reported | Not reported | Not reported | yes | Fetal chromosome abnormality, fetal malformation, IUGR, multiple pregnancies | Not reported |
| (61) | Suzuki 2016 | Not reported | Not clear | Not reported | Not reported | Not reported | Not reported | Not reported | Not reported | Not reported |
| (62) | White 2016 | yes | Not reported | Not reported | Not reported | Not reported | Not reported | Not reported | Not reported | First pregnancy, European descent |
| (63) | Ye 2016 | Not reported | yes | Not reported | Not reported | Not reported | Not reported | Not reported | Not reported | Chinese Hans |
| (65) | Herzog 2017 | no | Not reported | Not reported | Not reported | Not reported | Not reported | Not reported | HIV infected, aged <18 years, not able to read and understand the Dutch language, multiple birth pregnancies, pregnancies complicated by fetal congenital malformations | Not reported |
| (66) | Jia 2017 | Not reported | Not reported | Not reported | Not reported | Not reported | Not reported | Not reported | Spontaneous abortion, ectopic pregnancy, preterm delivery, or stillbirth | Caesarean section delivery |
| (67) | Kim 2017 | Not reported | yes | Not reported | yes | Not reported | Not reported | yes | Multiple gestations, gestational hypertension without proteinuria, nephritic syndrome diagnosed postpartum or other major metabolic disorders | Singleton pregnancies who received regular antenatal care at the prenatal care unit |
| (68) | Liu 2017a | yes | yes | Not reported | yes | yes | Not reported | Not reported | Intrapartum infection, gestational diabetes, chrioamnionitis, impulsive tearing of the membranes, alcohol abuse, long-term utilization of medicines, assisted reproduction | Singleton pregnancy |
| (69) | Liu 2017b | yes | yes | Not reported | Not reported | yes | Not reported | Not reported | Alcohol abuse, long-term addiction to medicines, assisted reproduction, gestational diabetes | Not reported |
| (70) | Majchrzak-Celinska 2017 | Not reported | Not reported | Not reported | Not reported | Not reported | Not reported | Not reported | Stillbirth, endocrine disease (except for hypothyroidism), liver disease, mental disorders, chronic infectious diseases | Singleton pregnancy, Polish Caucasian origin |
| (71) | Sari 2017 | Not reported | yes | Not reported | yes | yes | Not reported | Not reported | Proteinuria | Not reported |
| (72) | Van den Berg 2017 | no | Not reported | Not reported | Not reported | Not reported | Not reported | Not reported | Not reported | Not reported |
| (73) | Xiao 2017 | Not reported | yes | Not reported | yes | yes (chronic nephritis) | Not reported | Not reported | Systemic lupus erythematosus | Han ethnicity receiving caesarean section |
| (75) | Rahat 2017 | Not reported | Not reported | Not reported | Not reported | Not reported | yes | Not reported | IUGR, gestational trophoblast disease, gestational diabetes mellitus, Rh- isoimmunised women, thalassemia | Singleton pregnancies |
| (76) | Saraswathy 2017 | Not reported | yes | Not reported | Not reported | Not reported | yes | Not reported | Previous miscarriages, placental disorders, proteinuria and gestational diabetes mellitus | Singleton pregnancies of gestational age 21-40 gw without any systemic/infectious disease |
| (77) | Ma 2018 | Not reported | Not reported | Not reported | Not reported | Not reported | Not reported | Not reported | Serious maternal complications and fetal abnormalities | 3^rd^ trimester pregnant women |
| (78) | Mohammadpour-Gharehbagh 2018 | Not reported | yes | Not reported | yes | yes | Not reported | Not reported | Twin and multiple pregnancies, hydrops fetalis, liver dysfunction, all systemic diseases | Not reported |
| (79) | Rezaei 2018 | Not reported | yes | Not reported | yes | yes | Not reported | Not reported | Hydrops fetalis, twin or multiple pregnancies, liver dysfunction, all systemic diseases | Not reported |
| (80) | Wilson 2018 | Not reported | Not reported | Not reported | Not reported | Not reported | Not reported | Not reported | Multiple pregnancies, fetal and/or placental chromosomal abnormalities | Not reported |
| (83) | Zhu 2018 | Not reported | yes | yes (cardiac insufficiency) | yes | yes | Not reported | Not reported | Multiple pregnancies, HIV infection | Caesarean section |
| (84) | Zhang 2019 | yes | Not reported | Not reported | Not reported | Not reported | Not reported | Not reported | Pregnancy complications such as gestational diabetes mellitus, chronic hypertension, thyroid dysfunction, and kidney disease: alcohol abuse, assisted reproduction and  multiple pregnancies | Not reported |
| (85) | Fan 2019 | Not reported | Not reported | Not reported | Not reported | Not reported | Not reported | Not reported | Not reported | Vaginal deliveries |
| (86) | Gao 2019 | Not reported | yes (essential HTN) | yes | yes | yes | Not reported | Not reported | Not reported | Not reported |
| (87) | Halvatsiotis 2019 | Not reported | Not reported | Not reported | Not reported | Not reported | Not reported | Not reported | Alcohol and/or drug abuse during the current pregnancy, malignancies, infectious, chronic diseases | Below 40 years |
| (89) | Wang 2019 | Not reported | Not reported | Not reported | Not reported | Not reported | Not reported | Not reported | Not reported | Han ethnicity, caesarean section |

yes - indicates that women with the condition listed were excluded; no - indicates that women with the condition listed were included in the study; unclear – indicates that there are disagreement in statements about the examined criteria

^*^References number [1,2,3,4,6,16–18,26,29](32,37,81,82,88,90,38,44,51,52,57,59,64,74)(81,82,88,90) didn`t report exclusion criteria

Abbreviations: GDM – gestational diabetes mellitus; HTN – hypertension; IUGR – intrauterine growth restriction; GTD – gestational trophoblastic disease; CPM16 - Confined placental trisomy 16 mosaicism

Table S5. Differentially methylated genes in not specified PE

| Gene | No of CpGs | Region | hypo/hyper | tissue | Ref no |
| --- | --- | --- | --- | --- | --- |
| CYP27B1 | / | / | hyper | placenta | 41 |
| VDR | / | promoter | hyper | placenta | 41 |
| RXR | / | / | hyper | placenta | 41 |
| CPLX2 | / | TSS500, 5`-UTR | not sig; hypo | placenta; WBC | 28 |
| KIAA1609 | / | body | not sig; hypo | placenta; WBC | 28 |
| MFAP2 | / | TSS500, TSS1500 | not sig; hypo | placenta; WBC | 28 |
| SERPINA9 | / | TSS500, TSS1500 | hyper; hypo | placenta; WBC | 28 |
| SERPINA5 | / | TSS1500 | not sig; not sig | placenta; WBC | 28 |
| CD80 | / | TSS1500 | not sig; hypo | placenta; WBC | 28 |
| KRT23 | / | TSS1500 | not sig; hypo | placenta; WBC | 28 |
| PKHD1 | / | TSS500, TSS1500 | not sig; hypo | placenta; WBC | 28 |
| STMN2 | / | body | not sig; hypo | placenta; WBC | 28 |
| RAP1A | / | TSS1500 | not sig; hypo | placenta; WBC | 28 |
| UBE2G2 | / | 5`-UTR, body, TSS1500 | not sig; hypo | placenta; WBC | 28 |
| KHDC1 | / | TSS1500 | not sig; hypo | placenta; WBC | 28 |
| PLEKHA2 | / | 5`-UTR | not sig; hypo | placenta; WBC | 28 |
| LPL | 4 | promoter | not sig | placenta | 42 |
| SERPINB5 | 21 | promoter | not sig | placenta | 4 |
| RASSF1A | 22 16 / 4 / /  / | promoter promoter / promoter promoter /  promoter | not sig not sig hyper; hyper not sig; hypo not sig hyper  hyper | placenta  placenta  placenta; plasma  placenta; WBC  placenta  plasma  plasma | 4  47  21  59  3  8  76 |
| ICR1 | 2 | / | not sig | placenta | 5 |
| ICR2 | 7 | / | not sig | placenta | 5 |
| H19 | / | promoter | not sig | placenta | 5 |
| CDKN1C | 13 | promoter | not sig | placenta | 5 |
| PEG10 | / | promoter | not sig | placenta | 5 |
| PLAGL1 | / | promoter | not sig | placenta | 5 |
| SNRPN | / | promoter | not sig | placenta | 5 |
| MEST | / | exon 1 | not sig | placenta | 5 |
| LINE-1 | 7  4 | /  / | not sig  not sig | placenta  placenta | 5  70 |
| SERPINA3 | 14 | promoter | hypo | placenta | 40 |
| PAPPA2 | 1 | / | hyper | placenta | 40 |
| PSMD8 | 1 | / | hypo | placenta | 40 |
| NFKB2 | 1 | / | hypo | placenta | 40 |
| GRB2 | 1 | / | hyper | placenta | 40 |
| FLJ45983 | 1 | / | hypo | placenta | 30 |
| CDKN2A | 1 | / | hypo | placenta | 30 |
| POLD4 | 1 | / | hypo | placenta | 30 |
| CDKN2A | 1 | / | hypo | placenta | 30 |
| LAX1 | 1 | / | hypo | placenta | 30 |
| BCDIN3 | 1 | / | hypo | placenta | 30 |
| CCRL2 | 1 | / | hypo | placenta | 30 |
| RPS2 | 1 | / | hypo | placenta | 30 |
| KLHL21 | 1 | / | hypo | placenta | 30 |
| ELA2B | 1 | / | hypo | placenta | 30 |
| TSPAN18 | 1 | / | hyper | placenta | 30 |
| CEACAM7 | 1 | / | hyper | placenta | 30 |
| GATA5 | 1 | / | hyper | placenta | 30 |
| MIR34-A | / / | promoter promoter | hypo hypo | placenta  placenta | 44  79 |
| MTHFR | / /  17 | promoter promoter  promotor | hyper; hyper not sig  hyper; hypo | placenta; plasma  placenta  WBC; placenta | 45  38  88 |
| LEP | 62 | promoter | hypo | placenta | 25 |
| 11β-HSD2 | 49 | promoter | not sig | placenta | 46 |
| CAPN2 | 30 | promoter | hypo | placenta | 13 |
| EPHX2 | 12 6 | promoter promoter | hypo hypo | placenta  lymphocytes | 13 71 |
| ADORA2B | 32 | promoter | hypo | placenta | 13 |
| SOX7 | 22 | promoter | hypo | placenta | 13 |
| CXCL1 | 46 | promoter | hypo | placenta | 13 |
| CDX1 | 33 | promoter | hypo | placenta | 13 |
| IGFBP5 | 11 | promoter | hypo | placenta | 66 |
| DSCR3 | 12 | body | not sig | placenta | 47 |
| SOD1 | 4 | body | not sig | placenta | 47 |
| C2CD2 | 11 | body | not sig | placenta | 47 |
| UMODUL1 | 6 | body | not sig | placenta | 47 |
| ENST00000433952:-7924~-7988 | 8 | promoter | not sig | placenta | 47 |
| TERT  hTERT | 1  / | /  promoter | hyper; hyper  not sig; not sig | placenta; plasma  placenta; WBC | 55  39 |
| HLA-DOB | 1 | body | hyper; hyper | placenta; plasma | 55 |
| ACAP1 | 1 | / | hyper; hyper | placenta; plasma | 55 |
| SSTR5 | 1 | / | hyper; hyper | placenta; plasma | 55 |
| NBL1 | 1 | / | hyper; hyper | placenta; plasma | 55 |
| LRRTM4 | 1 | body | hyper; hyper | placenta; plasma | 55 |
| NTN4 | 1 | / | hyper; hyper | placenta; plasma | 55 |
| TRPS1 | 1 | body | hyper; hyper | placenta; plasma | 55 |
| ITPKB | 1 | TSS1500 | hyper; hyper | placenta; plasma | 55 |
| HECW1 | 1 | body | hyper; hyper | placenta; plasma | 55 |
| MIR193B | 1 | TSS1500 | hypo; hypo | placenta; plasma | 55 |
| GRK5 | 1 | body | hypo; hypo | placenta; plasma | 55 |
| SYDE1 | 1 | / | hypo; hypo | placenta; plasma | 55 |
| GRM3 | 1 | body | hypo; hypo | placenta; plasma | 55 |
| BAI1 | 1 | body | hypo; hypo | placenta; plasma | 55 |
| LINC00871 | 1 | body | hypo; hypo | placenta; plasma | 55 |
| ADARB2 | 1 | body | hypo; hypo | placenta; plasma | 55 |
| CDH4 | 1 | body | hypo; hypo | placenta; plasma | 55 |
| KRTI5 | 1 | TSS1500 | hypo; hypo | placenta; plasma | 55 |
| MIR137HG | 1 | body | hypo; hypo | placenta; plasma | 55 |
| RESISTIN | / | / | hypo | placenta | 32 |
| GLUT3 | / | / | hyper | placenta | 32 |
| RBP4 | / | / | hypo | placenta | 32 |
| PPARα | / | / | not sig | placenta | 32 |
| MASPIN | 13 | promoter | hypo | placenta | 33 |
| TF | / | promoter | hypo | placenta | 68 |
| WNT2 | / / | promoter TSS | hyper hyper | placenta  placenta | 69 64 |
| WNT2 | / | TSS | hyper | placenta | 64 |
| IGF1 | 1 | promoter | hyper | placenta | 77 |
| GATAD1 | 9 | 3`UTR | hypo | placenta | 35 |
| HSD11B2 | 1 | promoter | hypo | placenta | 70 |
| RUNX3 | 6 | promoter | not sig | placenta | 70 |
| MMP1 | 1 | promoter | hypo | omental fat arterie | 14 |
| TBXAS1 | / | / | hypo | omental fat arterie | 16 |
| APC | 11 | promoter | hypo; not sig | placenta; WBC | 59 |
| TIMP-3 | 57 | promoter | hypo | placenta | 25 |
| MMP9 | / 7 | promoter promoter | hyper; not sig hypo | placenta; WBC  placenta | 57 6 |
| TIMP-1 | 1 | promoter | not sig | placenta; WBC | 57 |
| STAT5A | 6 | promoter | hypo | plasma | 58 |
| PRKCDBP | 13 | promoter | hyper; not sig | placenta; WBC | 59 |
| P16 | 7 | promoter | hypo; hypo | placenta; WBC | 59 |
| RB1 | 16 | promoter | hypo; hypo | placenta; WBC | 59 |
| ERVW-1 | 20 | promoter | hyper | placenta | 22 |
| LAMA4 | 33 | promoter | not sig | placenta | 50 |
| HLA-G | 4 | promoter | hyper | placenta | 52 |
| LGALS13 | 1 | promoter | hypo | placenta | 37 |
| LGALS16 | 2 | promoter | hyper | placenta | 37 |
| LGALS14 | 3 | promoter | not sig | placenta | 37 |
| C-MYC | / | promoter | hyper; hypo | placenta; WBC | 39 |
| VEGF | / | promoter | hyper; not sig | placenta; WBC | 75 |
| EGFR | / | promoter | not sig; not sig | placenta; WBC | 75 |
| C-JUN | / | promoter | hyper; hyper | placenta; WBC | 75 |
| CD39 | 10 | body | 2 CpGs hypo | placenta | 83 |
| ZDHHC14 | 24 | body | 7 CpGs hypo | placenta | 83 |
| OXTR | 22  22 | Promoter  Promoter | hyper  hyper | Placental vessels  Umbilical vein | 85  86 |
| GRIN2B | 1 | / | hyper | MPB | 24 |
| SNX19 | 1 | / | hyper | MPB | 24 |
| PCDHB7 | 1 | / | hyper | MPB | 24 |
| CXX1 | 1 | / | hyper | MPB | 24 |
| BEX1 | 1 | / | hyper | MPB | 24 |
| MLLT6 | 1 | / | hyper | MPB | 24 |
| MTCP1 | 1 | / | hyper | MPB | 24 |
| C9orf23 | 1 | / | hyper | MPB | 24 |
| CCL26 | 2 | / | hyper | MPB | 24 |
| C11orf1 | 1 | / | hyper | MPB | 24 |
| FGF20 | 1 | / | hyper | MPB | 24 |
| TMEM100 | 1 | / | hyper | MPB | 24 |
| GABRA1 | 1 | / | hyper | MPB | 24 |
| RBBP7 | 1 | / | hyper | MPB | 24 |
| GM2A | 1 | / | hyper | MPB | 24 |
| C5orf15 | 1 | / | hyper | MPB | 24 |
| FTSJ3 | 1 | / | hyper | MPB | 24 |
| ZBP1 | 1 | / | hyper | MPB | 24 |
| POMC | 23 (3 sig) | shore | hypo | MPB | 62 |
| AGT | 8 (2 sig) | enhancer, 5` UTR | hyper | MPB | 62 |
| CALCA | 39 (2 sig) | enhancer | hyper; hypo | MPB | 62 |
| DDHA1 | 45 (2 sig) | body; shelf | hyper; hypo | MPB | 62 |
| SH3PXD2A | 88 | promoter | not sig | placenta | 26 |
| TFPI-2 | 31 | promoter | hypo | placenta | 73 |
| LAIR2 | / | / | hyper | placenta | 27 |
| CXCL1 | / | / | hyper | placenta | 27 |
| LAMA3 | / | / | hyper | placenta | 27 |
| LYZL1 | / | / | hyper | placenta | 27 |
| LY6K | / | / | hyper | placenta | 27 |
| MIG7 | / | / | hyper | placenta | 27 |
| CXCL2 | / | / | hyper | placenta | 27 |
| MADCAM1 | / | / | hyper | placenta | 27 |
| INGX | / | / | hyper | placenta | 27 |
| TXNDC6 | / | / | hyper | placenta | 27 |
| SGSM1 | / | / | hyper | placenta | 27 |
| OR51G1 | / | / | hyper | placenta | 27 |
| RNASE11 | / | / | hyper | placenta | 27 |
| C6orf105 | / | / | hyper | placenta | 27 |
| DNAJC5G | / | / | hyper | placenta | 27 |
| SSTR1 | / | / | hypo | placenta | 27 |
| SYT6 | / | / | hypo | placenta | 27 |
| LEMD1 | / | / | hypo | placenta | 27 |
| LOC641518 | / | / | hypo | placenta | 27 |
| TPSAB1 | / | / | hypo | placenta | 27 |
| ANKS1B | / | / | hypo | placenta | 27 |
| HCG26 | / | / | hypo | placenta | 27 |
| MXRA5 | / | / | hypo | placenta | 27 |
| TRIM31 | / | / | hypo | placenta | 27 |
| PRIMA1 | / | / | hypo | placenta | 27 |
| GTSF1 | / | / | hypo | placenta | 27 |
| RIPPLY1 | / | / | hypo | placenta | 27 |
| BTC | / | / | hypo | placenta | 27 |
| SLAMF1 | / | / | hypo | placenta | 27 |
| GNA12 | 8 | promoter | hypo; hypo | placenta; MPB | 63 |
| CBR1 | / | TSS, 5`-UTR, body | hyper | placenta | 64 |
| MIR548H4 | / | TSS, 5`-UTR, body | hyper | placenta | 64 |
| SPESP1 | / | TSS, 5`-UTR, body | hyper | placenta | 64 |
| NOX5 | / | TSS, 5`-UTR, body | hyper | placenta | 64 |
| MYH15 | / | body | hyper | placenta | 64 |
| CROT | / | TSS, 5`-UTR, body | hyper | placenta | 64 |
| TP53TG1 | / | TSS, 5`-UTR, body | hyper | placenta | 64 |
| GCSAML-AS1 | / | TSS, body, 3`-UTR | hyper | placenta | 64 |
| OR2C3 | / | TSS, body, 3`-UTR | hyper | placenta | 64 |
| FLJ41603 | / | TSS, 5`-UTR | hyper | placenta | 64 |
| WNT2 | / | TSS | hyper | placenta | 64 |
| CSNK1A1P | / | TSS, body | hyper | placenta | 64 |
| HOOK2 | / | body | hyper | placenta | 64 |
| FAM184B | / | body | hyper | placenta | 64 |
| LHX4 | / | body | hyper | placenta | 64 |
| HLA-DRB1 | / | body | hyper | placenta | 64 |
| ALCAM | / | body | hyper | placenta | 64 |
| SAR1B | / | TSS, 5`-UTR, body | hypo | placenta | 64 |
| NAPRT1 | / | TSS, 5`-UTR, body | hypo | placenta | 64 |
| SDK1 | / | body | hypo | placenta | 64 |
| CAPN8 | / | body | hypo | placenta | 64 |
| ARID1B | / | body | hypo | placenta | 64 |
| PURA | / | TSS< body, 3`-UTR | hypo | placenta | 64 |
| DIXDC1 | / | TSS, 5`-UTR, body | hypo | placenta | 64 |
| GPR75-ASB3 | / | TSS, 5`-UTR, body | hypo | placenta | 64 |
| ASB3 | / | TSS, 5`-UTR, body | hypo | placenta | 64 |
| CHAC2 | / | TSS, 5`-UTR, body | hypo | placenta | 64 |
| NKX1-2 | / | TSS, body | hypo | placenta | 64 |
| MST1P9 | / | body | hypo | placenta | 64 |
| ADORA2B | / | TSS, 5`-UTR, body | hypo | placenta | 64 |
| HKR1 | / | TSS, 5`-UTR, body, intragenic | hypo | placenta | 64 |
| MECOM | / | TSS, 5`-UTR, body | hypo | placenta | 64 |
| S-COMT | 14 | promoter | not sig; not sig; not sig | placenta; MPB; UCB | 8 |
| ITGB1 | 1 | / | hypo | placenta | 74 |
| PARP4 | 1 | / | hypo | placenta | 74 |
| ITGA5 | 2 | / | hypo | placenta | 74 |
| UBL3 | 1 | / | hypo | placenta | 74 |
| FN1 | 3 | / | hypo | placenta | 74 |
| ERRFI1 | 2 | / | hypo | placenta | 74 |
| BZW1 | 1 | / | hypo | placenta | 74 |
| KLF6 | 3 | / | hypo | placenta | 74 |
| FOS | 4 | / | hypo | placenta | 74 |
| TEAD1 | 1 | / | hypo | placenta | 74 |
| LDHA | 2 | / | hypo | placenta | 74 |
| DSC2 | 1 | / | hypo | placenta | 74 |
| NRIP1 | 1 | / | hypo | placenta | 74 |
| MALAT1 | 2 | / | hypo | placenta | 74 |
| LOC374443 | 1 | / | hypo | placenta | 74 |
| DUSP1 | 1 | / | hypo | placenta | 74 |
| REEP3 | 1 | / | hypo | placenta | 74 |
| UBASH3B | 1 | / | hypo | placenta | 74 |
| FLNB | 1 | / | hypo | placenta | 74 |
| TMEM65 | 1 | / | hypo | placenta | 74 |
| S100A10 | 1 | / | hypo | placenta | 74 |
| HTRA1 | 1 | / | hypo | placenta | 74 |
| MAN1A1 | 1 | / | hypo | placenta | 74 |
| ABCA1 | 1 | / | hypo | placenta | 74 |
| SHANK3 | 2 | / | hyper | placenta | 74 |
| ARHGAP17 | 1 | / | hyper | placenta | 74 |
| IFITM5 | 1 | / | hyper | placenta | 74 |
| BAX | / | promoter | not sig | placenta | 90 |
| BCL2 | / | promoter | not sig | placenta | 90 |
| ATF2 | / | / | not sig | Maternal peripheral blood | 87 |
| CCL25 | / | / | hypomethylation | Maternal peripheral blood | 87 |
| CXCL14 | / | / | hypomethylation | Maternal peripheral blood | 87 |
| CXCL3 | / | / | hypomethylation | Maternal peripheral blood | 87 |
| CXCL5 | / | / | hypomethylation | Maternal peripheral blood | 87 |
| CXCL6 | / | / | hypomethylation | Maternal peripheral blood | 87 |
| IL10RA | / | / | not sig | Maternal peripheral blood | 87 |
| IL12A | / | / | not sig | Maternal peripheral blood | 87 |
| IL12B | / | / | hypomethylation | Maternal peripheral blood | 87 |
| IL13 | / | / | not sig | Maternal peripheral blood | 87 |
| IL13RA1 | / | / | hypomethylation | Maternal peripheral blood | 87 |
| IL17C | / | / | hypomethylation | Maternal peripheral blood | 87 |
| IL6ST | / | / | hypomethylation | Maternal peripheral blood | 87 |
| IL17RA | / | / | hypomethylation | Maternal peripheral blood | 87 |
| IL4R | / | / | not sig | Maternal peripheral blood | 87 |
| IL6R | / | / | not sig | Maternal peripheral blood | 87 |
| INHA | / | / | hypomethylation | Maternal peripheral blood | 87 |
| IL7 | / | / | hypomethylation | Maternal peripheral blood | 87 |
| AVPR1 | 14 | promoter | hypermethylation | umbilical vein | 86 |
| PKCB | 44 | promoter | hypermethylation | umbilical vein | 86 |

References:

1. Müller HM, Ivarsson L, Schröcksnadel H, et al. DNA methylation changes in sera of women in early pregnancy are similar to those in advanced breast cancer patients.Clin Chem. 2004;50(6):1065‐1068.

2. Chelbi ST, Mondon F, Jammes H, et al. Expressional and epigenetic alterations of placental serine protease inhibitors: SERPINA3 is a potential marker of preeclampsia. Hypertension. 2007;49(1):76–83.

3. Tsui DW, Chan KC, Chim SS et al. Quantitative aberrations of hypermethylated RASSF1A gene sequences in maternal plasma in pre-eclampsia. Prenat Diagn. 2007;27(13):1212–8.

4. Bellido ML, Radpour R, Lapaire O, et al. MALDI-TOF Mass Array Analysis of RASSF1A and SERPINB5 Methylation Patterns in Human Placenta and Plasma1. Biol Reprod. 2010;82(4):745–750.

5. Bourque DK, Avila L, Peñaherrera M, von Dadelszen P, Robinson WP. Decreased Placental Methylation at the H19/IGF2 Imprinting Control Region is Associated with Normotensive Intrauterine Growth Restriction but not Preeclampsia. Placenta. 2010;31(3):197–202.

6. Wang Z, Lu S, Liu C, et al. Expressional and epigenetic alterations of placental matrix metalloproteinase 9 in preeclampsia. Gynecol Endocrinol. 2010;26(2):96–102.

7. Yuen RK, Pẽaherrera MS, von Dadelszen P, McFadden DE, Robinson WP. DNA methylation profiling of human placentas reveals promoter hypomethylation of multiple genes in early-onset preeclampsia. Eur J Hum Genet. 2010;18(9):1006–1012.

8. Zhao F, Wang J, Liu R, et al. Quantification and application of the placental epigenetic signature of the RASSF1A gene in maternal plasma. Prenat Diagn 2010;30(8):778–782.

9. Chelbi ST, Doridot L, Mondon F, et al. Combination of promoter hypomethylation and PDX1 overexpression leads to TBX15 decrease in vascular IUGR placentas. Epigenetics. 2011;6(2):247–255.

10. Gao WL, Li D, Xiao ZX, et al. Detection of global DNA methylation and paternally imprinted H19 gene methylation in preeclamptic placentas. Hypertens Res. 2011;34(5):655–661.

11. Kulkarni A, Chavan-Gautam P, Mehendale S, Yadav H, Joshi S. Global DNA methylation patterns in placenta and its association with maternal hypertension in Pre-eclampsia. DNA Cell Biol. 2011;30(2):79–84.

12. Zhao A, Cheng Y, Li X, et al. Promoter hypomethylation of COMT in human placenta is not associated with the development of pre-eclampsia. Mol Hum Reprod. 2011;17(3):199–206.

13. Jia RZ, Zhang X, Hu P, et al. Screening for differential methylation status in human placenta in preeclampsia using a CpG island plus promoter microarray. Int J Mol Med. 2012;30(1):133–141.

14. Mousa AA, Archer KJ, Cappello R, et al. DNA methylation is altered in maternal blood vessels of women with preeclampsia. Reprod Sci. 2012;19(12):1332–1342.

15. Mousa AA, Cappello RE, Estrada-Gutierrez G, et al. Preeclampsia is associated with alterations in DNA methylation of genes involved in collagen metabolism. Am J Pathol. 2012;181(4):1455–1463.

16. Mousa AA, Strauss JF 3rd, Walsh SW. Reduced Methylation of the Thromboxane Synthase Gene Is Correlated With Its Increased Vascular Expression in Preeclampsia. Hypertension. 2012;59(6):1249-1255.

17. Blair JD, Yuen RK, Lim BK, McFadden DE, von Dadelszen P, Robinson WP. Widespread DNA hypomethylation at gene enhancer regions in placentas associated with early-onset pre-eclampsia. Mol Hum Reprod. 2013;19(10):697–708.

18. Czikk MJ, Drewlo S, Baczyk D, Adamson SL, Kingdom J. Dual specificity phosphatase 9 (DUSP9) expression is down-regulated in the severe pre-eclamptic placenta. Placenta. 2013;34(2):174–181.

19. Hogg K, Blair JD, McFadden DE, von Dadelszen P, Robinson WP. Early Onset Pre-Eclampsia Is Associated with Altered DNA Methylation of Cortisol-Signalling and Steroidogenic Genes in the Placenta. PLoS One. 2013;8(5):e62969.

20. Hogg K, Blair JD, von Dadelszen P, Robinson WP. Hypomethylation of the LEP gene in placenta and elevated maternal leptin concentration in early onset pre-eclampsia. Mol Cell Endocrinol. 2013;367(1–2):64–73.

21. Kim MJ, Kim SY, Park SY, Ahn HK, Chung JH, Ryu HM. Association of fetal-derived hypermethylated RASSF1A concentration in placenta-mediated pregnancy complications. Placenta. 2013;34(1):57–61.

22. Ruebner M, Strissel PL, Ekici AB, et al. Reduced Syncytin-1 Expression Levels in Placental Syndromes Correlates with Epigenetic Hypermethylation of the ERVW-1 Promoter Region. PLoS One. 2014;9(8):e107215].PLoS One. 2013;8(2):e56145.

23. Sundrani DP, Reddy US, Joshi AA, et al. Differential placental methylation and expression of VEGF, FLT-1 and KDR genes in human term and preterm preeclampsia.Clin Epigenetics. 2013;5(1):6.

24. White WM, Brost B, Sun Z, et al. Genome-wide methylation profiling demonstrates hypermethylation in maternal leukocyte DNA in preeclamptic compared to normotensive pregnancies. Hypertens Pregnancy. 2013;32(3):257–269.

25. Xiang Y, Cheng Y, Li X, et al. Up-Regulated Expression and Aberrant DNA Methylation of LEP and SH3PXD2A in Pre-Eclampsia. PLoS One. 2013;8(3):e59753.

26. Xiang Y, Zhang X, Li Q, et al. Promoter hypomethylation of TIMP3 is associated with pre-eclampsia in a Chinese population. Mol Hum Reprod. 2013;19(3):153–159.

27. Yan YH, Yi P, Zheng YR, et al. Screening for preeclampsia pathogenesis related genes.Eur Rev Med Pharmacol Sci. 2013;17(22):3083-3094.

28. Anderson CM, Ralph JL, Wright ML, Linggi B, Ohm JE. DNA Methylation as a Biomarker for Preeclampsia. Biol Res Nurs. 2014;16(4):409–420.

29. Anton L, Brown AG, Bartolomei MS, Elovitz MA. Differential methylation of genes associated with cell adhesion in preeclamptic placentas. PLoS One. 2014;9(6):e100148.

30. Chu T, Bunce K, Shaw P, et al. Comprehensive analysis of preeclampsia-associated DNA methylation in the placenta. PLoS One. 2014;9(9):e107318.

31. Liu H, Tang Y, Liu X, et al. 14-3-3 tau (YWHAQ) gene promoter hypermethylation in human placenta of preeclampsia. Placenta. 2014;35(12):981–988.

32. Liu L, Zhang X, Rong C, et al. Distinct DNA methylomes of human placentas between pre-eclampsia and gestational diabetes mellitus. Cell Physiol Biochem. 2014;34(6):1877–1889.

33. Liu Q, Qiao FY, Shi XW, Liu HY, Gong X, Wu YY. Promoter hypomethylation and increased maspin expression in preeclamptic placentas in a Chinese population. Placenta. 2014;35(11):876–882.

34. Lu L, Hou Z, Li L, et al. Methylation pattern of H19 exon 1 is closely related to preeclampsia and trophoblast abnormalities. Int J Mol Med. 2014;34(3):765–771.

35. Ma X, Li J, Brost B, Cheng W, Jiang SW. Decreased expression and DNA methylation levels of GATAD1 in preeclamptic placentas. Cell Signal. 2014;26(5):959–967.

36. Nomura Y, Lambertini L, Rialdi A, et al. Global methylation in the placenta and umbilical cord blood from pregnancies with maternal gestational diabetes, preeclampsia, and obesity. Reprod Sci. 2014;21(1):131–137.

37. Than NG, Romero R, Xu Y, et al. Evolutionary origins of the placental expression of chromosome 19 cluster galectins and their complex dysregulation in preeclampsia.Placenta. 2014;35(11):855-865.

38. Blair JD, Langlois S, McFadden DE, Robinson WP. Overlapping DNA methylation profile between placentas with trisomy 16 and early-onset preeclampsia. Placenta. 2014;35(3):216–222.

39. Rahat B, Hamid A, Ahmad Najar R, Bagga R, Kaur J. Epigenetic mechanisms regulate placental c-myc and hTERT in normal and pathological pregnancies; c-myc as a novel fetal DNA epigenetic marker for pre-eclampsia.Mol Hum Reprod. 2014;20(10):1026-1040.

40. Ching T, Song MA, Tiirikainen M, et al. Genome-wide hypermethylation coupled with promoter hypomethylation in the chorioamniotic membranes of early onset pre-eclampsia. Mol Hum Reprod. 2014;20(9):885–904.

41. Anderson CM, Ralph JL, Johnson L, et al. First trimester vitamin D status and placental epigenomics in preeclampsia among Northern Plains primiparas. Life Sci. 2015;129:10–15.

42. Barrett HL, Kubala MH, Scholz Romero K, et al. Placental lipase expression in pregnancies complicated by preeclampsia: A case-control study. Reprod Biol Endocrinol. 2015;13:100.

43. Ching T, Ha J, Song MA, et al. Genome-scale hypomethylation in the cord blood dnas associated with early onset preeclampsia. Clin Epigenetics. 2015;7(1):21.

44. Doridot L, Houry D, Gaillard H, Chelbi ST, Barbaux S, Vaiman D. miR-34A expression, epigenetic regulation, and function in human placental diseases. Epigenetics. 2014;9(1):142–151.

45. Ge J, Wang J, Zhang F, et al. Correlation between MTHFR gene methylation and pre-eclampsia, and its clinical significance. Genet Mol Res. 2015;14(3):8021–8028.

46. Hu W, Wang H, Huang H. Analysis of gene expression and preliminary study of methylation about 11β-HSD2 gene in placentas of Chinese pre-eclampsia patients of Han ethnicity. J Obstet Gynaecol Res. 2015;41(3):343–349.

47. Kim HJ, Kim SY, Lim JH, Kwak DW, Park SY, Ryu HM. Quantification and application of potential epigenetic markers in maternal plasma of pregnancies with hypertensive disorders. Int J Mol Sci. 2015;16(12):29875–29888.

48. Martin E, Ray PD, Smeester L, Grace MR, Boggess K, Fry RC. Epigenetics and preeclampsia: Defining functional epimutations in the preeclamptic placenta related to the TGF-β pathway. PLoS One. 2015;10(10):e0141294.

49. Qi YH, Teng F, Zhou Q, et al. Unmethylated-maspin DNA in maternal plasma is associated with severe preeclampsia. Acta Obstet Gynecol Scand. 2015;94(9):983–988.

50. Shan N, Zhang X, Xiao X, et al. Laminin α4 (LAMA4) expression promotes trophoblast cell invasion, migration, and angiogenesis, and is lowered in preeclamptic placentas. Placenta. 20151;36(8):809–820.

51. Shimanuki Y, Mitomi H, Fukumura Y, et al. Alteration of Delta-like ligand 1 and Notch 1 receptor in various placental disorders with special reference to early onset preeclampsia. Hum Pathol. 2015;46(8):1129–1137.

52. Tang Y, Liu H, Li H, Peng T, Gu W, Li X. Hypermethylation of the HLA-G promoter is associated with preeclampsia. Mol Hum Reprod. 2015;21(9):736–744.

53. Zhu L, Lv R, Kong L, Cheng H, Lan F, Li X. Genome-wide mapping of 5mC and 5hmC identified differentially modified genomic regions in late-onset severe preeclampsia: A pilot study. PLoS One. 2015;10(7):e0134119.

54. Xuan J, Jing Z, Yuanfang Z, et al. Comprehensive analysis of DNA methylation and gene expression of placental tissue in preeclampsia patients. Hypertens Pregnancy. 2016;35(1):129–138.

55. Kim JH, Cheong HS, Lee DS, Shin HD, Kim YN. Genome-wide DNA methylation profiles of maternal peripheral blood and placentas: potential risk factors for preeclampsia and validation of GRK5. Genes and Genomics. 2017;39(2):197–206.

56. Lin L, Yu Y, Zhang Z, Yang Y. Significant hypomethylation of TNFAIP8 and increased expression in the placenta and peripheral blood cells from early-onset preeclamptic patients. Int J Clin Exp Med. 2016.9(6):10384-10393.

57. Rahat B, Sharma R, Bagga R, Hamid A, Kaur J. Imbalance between matrix metalloproteinases and their tissue inhibitors in preeclampsia and gestational trophoblastic diseases. Reproduction. 2016;152(1):11–22.

58. Rahat B, Thakur S, Bagga R, Kaur J. Epigenetic regulation of STAT5A and its role as fetal DNA epigenetic marker during placental development and dysfunction. Placenta. 2016;44:46–53.

59. Rahat B, Thakur S, Hamid A, Bagga R, Kaur J. Association of aberrant methylation at promoter regions of tumor suppressor genes with placental pathologies. Epigenomics. 2016;8(6):767–787.

60. Wilson SL, Liu Y, Robinson WP. Placental telomere length decline with gestational age differs by sex and TERT, DNMT1, and DNMT3A DNA methylation. Placenta. 2016;48:26–33.

61. Suzuki M, Maekawa R, Patterson NE, et al. Amnion as a surrogate tissue reporter of the effects of maternal preeclampsia on the fetus. Clin Epigenetics. 2016;8:67.

62. White WM, Sun Z, Borowski KS, et al. Preeclampsia/Eclampsia candidate genes show altered methylation in maternal leukocytes of preeclamptic women at the time of delivery. Hypertens Pregnancy. 2016;35(3):394–404.

63. Ye W, Shen L, Xiong Y, Zhou Y, Gu H, Yang Z. Preeclampsia is Associated with Decreased Methylation of the GNA12 Promoter. Ann Hum Genet. 2016;80(1):7–10.

64. Yeung KR, Chiu CL, Pidsley R, Makris A, Hennessy A, Lind JM. DNA methylation profiles in preeclampsia and healthy control placentas. Am J Physiol Hear Circ Physiol. 2016;310(10):H1295-H1303.

65. Herzog EM, Eggink AJ, Willemsen SP, et al. Early- and late-onset preeclampsia and the tissue-specific epigenome of the placenta and newborn. Placenta. 2017;58:122–132.

66. Jia Y, Li T, Huang X, et al. Dysregulated DNA Methyltransferase 3A Upregulates IGFBP5 to Suppress Trophoblast Cell Migration and Invasion in Preeclampsia. Hypertension. 2017;69(2):356–366.

67. Kim SY, Kim HJ, Park SY, Han YJ, Choi JS, Ryu HM. Early Prediction of Hypertensive Disorders of Pregnancy Using Cell-Free Fetal DNA, Cell-Free Total DNA, and Biochemical Markers. Fetal Diagn Ther. 2016;40(4):255–262.

68. Liu R, Ma Q, Wen A, et al. Increased tissue factor expression and promoter hypomethylation in preeclampsia placentas in a Chinese population. Pregnancy Hypertens. 2017;10:90–95.

69. Liu Y, Ma Y. Promoter methylation status of WNT2 in placenta from patients with preeclampsia. Med Sci Monit. 2017;23:5294–5301.

70. Majchrzak-Celińska A, Kosicka K, Paczkowska J, et al. HSD11B2, RUNX3, and LINE-1 Methylation in Placental DNA of Hypertensive Disorders of Pregnancy Patients. Reprod Sci. 2017;24(11):1520–1531.

71. Sari I, Pinarbasi H, Pinarbasi E, Yildiz C. Association between the soluble epoxide hydrolase gene and preeclampsia. Hypertens Pregnancy. 2017 ;36(4):315–325.

72. van den Berg CB, Chaves I, Herzog EM, Willemsen SP, van der Horst GTJ, Steegers-Theunissen RPM. Early- and late-onset preeclampsia and the DNA methylation of circadian clock and clock-controlled genes in placental and newborn tissues. Chronobiol Int. 2017;34(7):921–932.

73. Xiao X, Tao X, Wang Y, et al. Hypomethylation of tissue factor pathway inhibitor 2 in human placenta of preeclampsia. Thromb Res. 2017;152:7–13.

74. Zhao M, Li L, Yang X, Cui J, Li H. FN1, FOS, and ITGA5 induce preeclampsia: Abnormal expression and methylation. Hypertens Pregnancy. 2017;36(4):302–309.

75. Rahat B, Najar RA, Hamid A, Bagga R, Kaur J. The role of aberrant methylation of trophoblastic stem cell origin in the pathogenesis and diagnosis of placental disorders.Prenat Diagn. 2017;37(2):133-143.

76. Saraswathy S, Sahai K, Arora D, et al. Fetal-specific hypermethylated RASSF1A quantification in pregnancy. J Matern Neonatal Med. 2017;30(7):849–853.

77. Ma M, Zhou QJ, Xiong Y, Li B, Li XT. Preeclampsia is associated with hypermethylation of IGF-1 promoter mediated by DNMT1.Am J Transl Res. 2018;10(1):16‐39.

78. Mohammadpour-Gharehbagh A, Teimoori B, Narooei-nejad M, Mehrabani M, Saravani R, Salimi S. The association of the placental MTHFR 3′-UTR polymorphisms, promoter methylation, and MTHFR expression with preeclampsia. J Cell Biochem. 2018;119(2):1346–1354.

79. Rezaei M, Eskandari F, Mohammadpour-Gharehbagh A, Harati-Sadegh M, Teimoori B, Salimi S. Hypomethylation of the miRNA-34a gene promoter is associated with Severe Preeclampsia. Clin Exp Hypertens. 2019;41(2):118–122.

80. Wilson SL, Leavey K, Cox BJ, Robinson WP. Mining DNA methylation alterations towards a classification of placental pathologies. Hum Mol Genet. 2018;27(1):135–146.

81. Alahari S, Garcia J, Post M, Caniggia I. The von Hippel Lindau tumour suppressor gene is a novel target of E2F4-mediated transcriptional repression in preeclampsia. BiochimBiophys Acta Mol Basis Dis. 2018;1864(10):3298‐3308.

82. Li X, Wu C, Shen Y, et al. Ten-eleven translocation 2 demethylates the MMP9 promoter, and its down-regulation in preeclampsia impairs trophoblast migration and invasion. J Biol Chem. 2018;293(26):10059–10070.

83. Zhu L, Lv R, Kong L, Li X. Reduced methylation downregulates CD39/ENTPD1 and ZDHHC14 to suppress trophoblast cell proliferation and invasion: Implications in preeclampsia. Pregnancy Hypertens. 2018;14:59–67.

84. Zhang L, Leng M, Li Y, et al. Altered DNA methylation and transcription of WNT2 and DKK1 genes in placentas associated with early-onset preeclampsia. Clin Chim Acta. 2019;490:154–160.

85. Fan X, Xu T, Ding H, et al. DNA methylation-reprogrammed oxytocin receptor underlies insensitivity to oxytocin in pre-eclamptic placental vasculature. J Cell Mol Med. 2019;23(6):4118–4126.

86. Gao Q, Fan X, Xu T, et al. Promoter methylation changes and vascular dysfunction in pre-eclamptic umbilical vein. Clin Epigenetics. 2019;11(1):84.

87. Halvatsiotis P, Tsokaki T, Chrelias C, et al. Methylation profile of genes involved in inflammation, in the blood from pregnancies with maternal preeclampsia due to untreated gestational diabetes mellitus. Hormones. 2019;18(2):173–178.

88. Mishra J, Talwar S, Kaur L, et al. Differential global and MTHFR gene specific methylation patterns in preeclampsia and recurrent miscarriages: A case-control study from North India. Gene. 2019;704:68–73.

89. Wang T, Xiang Y, Zhou X, et al. Epigenome-wide association data implicate fetal/maternal adaptations contributing to clinical outcomes in preeclampsia. Epigenomics. 2019;11(9):1003–1019.

90. Mohammadpour-Gharehbagh A, jahantigh D, Eskandari M, et al. Genetic and epigenetic analysis of the BAX and BCL2 in the placenta of pregnant women complicated by preeclampsia. Apoptosis. 2019;24(3–4):301–311.
